# A Systematic Bayesian Integration of Epidemiological and Genetic Data

**DOI:** 10.1371/journal.pcbi.1004633

**Published:** 2015-11-23

**Authors:** Max S. Y. Lau, Glenn Marion, George Streftaris, Gavin Gibson

**Affiliations:** 1 Department of Ecology and Evolutionary Biology, Princeton, New Jersey, United States of America; 2 Biomathematics and Statistics Scotland, Edinburgh, United Kingdom; 3 Department of Actuarial Mathematics and Statistics, Heriot-Watt University, Edinburgh, United Kingdom; Duke University, UNITED STATES

## Abstract

Genetic sequence data on pathogens have great potential to inform inference of their transmission dynamics ultimately leading to better disease control. Where genetic change and disease transmission occur on comparable timescales additional information can be inferred via the joint analysis of such genetic sequence data and epidemiological observations based on clinical symptoms and diagnostic tests. Although recently introduced approaches represent substantial progress, for computational reasons they approximate genuine joint inference of disease dynamics and genetic change in the pathogen population, capturing partially the joint epidemiological-evolutionary dynamics. Improved methods are needed to fully integrate such genetic data with epidemiological observations, for achieving a more robust inference of the transmission tree and other key epidemiological parameters such as latent periods. Here, building on current literature, a novel Bayesian framework is proposed that infers simultaneously and explicitly the transmission tree and unobserved transmitted pathogen sequences. Our framework facilitates the use of realistic likelihood functions and enables systematic and genuine joint inference of the epidemiological-evolutionary process from partially observed outbreaks. Using simulated data it is shown that this approach is able to infer accurately joint epidemiological-evolutionary dynamics, even when pathogen sequences and epidemiological data are incomplete, and when sequences are available for only a fraction of exposures. These results also characterise and quantify the value of incomplete and partial sequence data, which has important implications for sampling design, and demonstrate the abilities of the introduced method to identify multiple clusters within an outbreak. The framework is used to analyse an outbreak of foot-and-mouth disease in the UK, enhancing current understanding of its transmission dynamics and evolutionary process.

## Author Summary

In the midst of increasingly available sequence data of pathogens, a key challenge is to better integrate these data with traditional epidemiological data, with the proximate goal of reliable prediction and the ultimate aim of effective management of disease outbreaks. Although substantial advances have been made for such an integration, and they have improved our understandings of many disease dynamics which are not available otherwise, current methods have relied on fast algorithms, rather than achieving a systematic integration and accurate inference of the joint epidemiological-evolutionary process. Building on methods in current literature, this paper describes a novel Bayesian approach for systematically integrating these two streams of data. We propose a computationally tractable Bayesian inferential algorithm which takes the full joint epidemiological-evolutionary process into account. Using this algorithm, we study systematically the value of genetic data, providing valuable insights into future sampling designs. The algorithm is subsequently applied to real-world dataset describing the spread of animal foot-and-mouth disease in the UK, demonstrating the importance of such a systematic integration achieved with our methodology.

## Introduction

Epidemiological data for infectious disease, defined here as clinical observation, diagnostic test results and associated covariates such as location, only indirectly reflect underlying contact structures, exposure times, and other aspects of disease dynamics. Developments in Bayesian data-augmentation methodology for spatio-temporal processes over the last decade or so [1–4] allow key epidemiological quantities, e.g. contact rates and latent periods, that are critical to risk assessment and disease control, to be inferred from such data. These methods typically employ stochastic integration techniques such as Markov Chain Monte Carlo (MCMC) to infer the full history of the epidemic, including the transmission tree, from partial observations. Unfortunately, epidemiological data available for an epidemic outbreak typically do not typically allow very precise inference of detailed aspects of disease transmission dynamics [5].

However, a parallel development is the increasing availability of genetic data on pathogens collected, in particular, based on whole genome sequencing [6–8]. During an outbreak pathogen populations are subject to genetic change through mutation and selection. Genetic data on pathogens, sampled from exposed hosts within an outbreak, therefore carry information on relatedness of different infection events. When genetic change and disease transmission occur on comparable time scales joint analysis of epidemiological and genetic data can lead to valuable insights concerning epidemic outbreaks. For example, it can help us to identify the transmission network [9] which can be used to quantify superspreading events [10], to study the evolutionary patterns of pathogens [11] and to design and evaluate of control measures [12].

**Fig 1:**
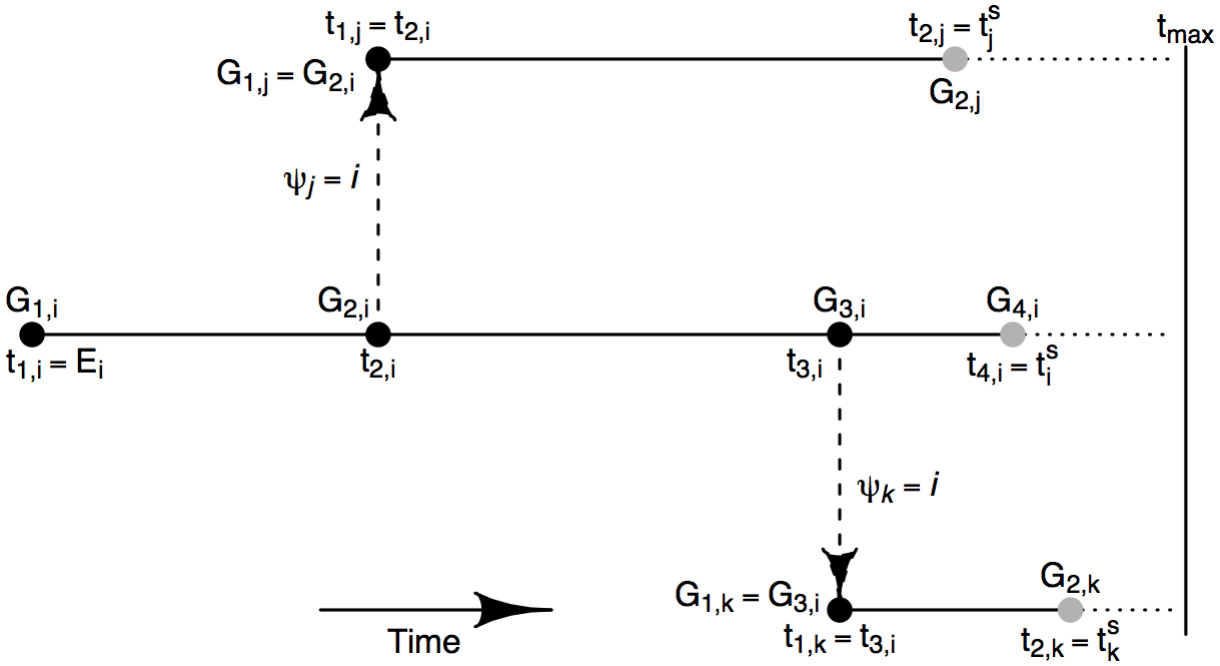
A sequence of events in which individual *i* infects individuals *j* and then *k* (dashed arrows) along with the sampling of sequences taken from these individuals. Solid circles represent the sequences at respective time points. Among these events only the sampling times $t_{i}^{s}$, $t_{j}^{s}$, $t_{k}^{s}$ and the corresponding sequence samples (coloured grey) are typically observed, while other unobserved quantities are to be imputed (see later). Other events potentially occurring on the dotted lines are not shown. Note that in our inference we do not demand that all exposures have an observed sequence. Also note that if individual *i* is a primary infection, *G*_1,*i*_ is assumed to be a stochastic variant of the universal master sequence *G*_*M*_ (see *Multiple and Single Primary Infection Model*).

Approaches that rely on reconstructing *phylogenetic trees* have been followed in several scenarios [13, 14]. A number of limitations of these approaches are highlighted in [15]. For example, when the sampled sequences include donor-recipient pairs with respect to the infection process, a situation commonly arising during the early stages of an epidemic, these approaches may not capture adequately the direct ancestor-descendant relationship between them. This paper presents novel methodology which advances the joint analysis of epidemiological and genetic data, building on recent substantial progress of others [16–21]. These authors sought to overcome the limitations noted above of using phylogenetic trees as a proxy for transmission dynamics, developing approaches which explicitly construct *transmission trees* by combining genetic and epidemiological data [16, 18–22]. These methods have proved to be very valuable in unravelling transmission paths during an epidemic outbreak. However, they employ various approximations/simplifications which either avoid explicit inference of the unobserved sequences from pathogens Transmitted from donors to recipients upon infection (solid black circles in Fig 1) [16, 18–21] or use approximate Bayesian inference to account for these sequences [22]. Thus they may not fully infer the entire epidemiological-evolutionary process and may not utilise the most appropriate likelihood function (see section *Complete-data Likelihood*). For example, [19] considers sequence combinations that exhibit the minimum amount of mutation necessary to explain sub-trees of transmission connecting the observed pathogen strains; [16, 20] consider a pseudo-likelihood computed for only observed sequences; and, as opposed to a genuine joint approach, [17] considers a two-step inference procedure, whereby a phylogeny is first constructed independently of the transmission network before conducting inference of the transmission network. These approximate approaches greatly reduce the computational challenges inherent in inferring the unobserved transmitted sequences, and facilitate statistical inference, particularly when the transmission tree is of primary interest. However, there is certainly scope for improving on their performance and better capturing the joint epidemiological-evolutionary dynamics. For example, it is already recognised that reconstruction of the transmission tree can be sensitive to the choice of prior for some epidemiological parameters [16], suggesting that a more rigorous joint inference may yield improved inference. In addition, the latent period of a disease may be overestimated by ignoring the unobserved pathogen sequences transmitted upon infections [20]. Further research on the systematic integration of epidemiological and genetic data, in the context of inferring both the transmission tree and the epidemiological-evolutionary process, is therefore warranted.

It is well-known, particularly within a Bayesian framework, that explicit imputation of unobserved processes is a beneficial strategy for addressing such issues. This enables the use of likelihood functions consistent with models that better represent the underlying processes e.g. reducing bias when quantifying disease dynamics from epidemiological data [23–26]. In this paper we therefore address the challenge of explicitly imputing transmitted sequences within the framework of data-augmented Bayesian analysis whereby unobserved processes are treated as supplementary unknown parameters. In the context of joint inference of epidemiological-evolutionary processes, the unobserved data include not only standard aspects related to epidemiological data, such as exposure times, but also unobserved genetic sequences transmitted during these events. Implementation of inference e.g. via MCMC, is accordingly more computationally challenging than for epidemic data only, due to the complexity of the data-augmented parameter space which comprises the model parameters and all potential transmission graphs and sequences consistent with the observed data.

Within the Bayesian framework the result of inference is described by the *posterior* distribution over data-augmented parameter space. MCMC algorithms draw correlated samples from the posterior which are used to generate statistics of interest e.g. the marginal posterior distribution of transmission trees. In this context Markov chains which produce highly correlated samples are described as poorly mixing. Standard MCMC algorithms, such as the single-component Metropolis-Hastings algorithm, make updates to a single model parameter at any time. However, for the problem that we consider here, identifying well-designed proposal schemes for jointly updating components is challenging, but necessary for obtaining a well-mixing Markov chain that can efficiently explore the joint posterior distribution of model parameters, transmission graphs and transmitted pathogen sequences. Specifically, the challenge arises when proposing updates to the source of a given infection. A naive algorithm may update the source of infection leaving the corresponding transmitted sequence unchanged so that the downstream pathogen sequences would still belong to the previous branch of the infection tree. It is easy to see that this would lead to a very low acceptance probability for the proposed change and inefficient exploration of the domain of transmission trees and sequences. A crucial research challenge, and key aim of this paper is therefore, to devise a computationally tractable algorithm for the joint proposal of unobserved sequences and the transmission tree to be embeded within an MCMC algorithm.

We also consider the general case of epidemics with arbitrary numbers of *clusters* (where a cluster is a set of infections arising from a single primary infection), of which the one-cluster scenario considered in many practical applications (e.g. [19, 20]) is a special case. In contrast to existing approaches [16, 18] to the multi-cluster scenario, we model explicitly the process of generating sequences for background/primary infections (see *Models and Methods*). Note that, when including multiple-cluster scenarios, a transmission *tree*, which is the term used routinely in the literature where typically a single cluster is assumed [19, 20], should be referred to as a transmission *graph* (or sometimes transmission *forest*). In summary the main outcomes reported in the paper are as follows.

1. We devise a statistically sound and computationally tractable Bayesian framework that facilitates *systematic* integration of epidemiological and genetic data. Specifically, we formulate Bayesian tools for imputing unobserved data, particularly for the joint proposal of the transmission graph and the sequences transmitted (at times of infection), facilitating a more explicit representation and accurate recovery of the processes of epidemic transmission and pathogen evolution, even when only data on a *subset* of the infected population are available.
2. Having enabled systematic integration of epidemiological and evolutionary process, we characterise and quantify systematically the importance of genetic data for the inference of some important aspects of epidemic dynamics: the inference of the transmission graph, epidemiological parameters and the identification of clusters. Moreover we demonstrate that genetic data may also facilitate model assessment using methods recently developed by the authors [27].
3. We demonstrate the reliability of these novel methods using simulated data and their practical utility by analysing a foot-and-mouth outbreak in the UK.

## Models

Technical details of our methods are presented in the following order. First, the specific details of the underlying epidemic process and a description of the representation of pathogen sequences and their evolution are given in sections *The Stochastic Epidemic Process* and *Stochastic Process for Genetic Evolution* respectively. Details of the primary infection model required to allow imputation of multiple clusters are given in section *Multiple and Single Primary Infection Model*, and these details are combined in the *Complete-data Likelihood*. The implementation of our novel inferential framework using partial observation of the processes described by this model is outlined in the section *A Systematic Bayesian Integration Framework*. In particular this section describes Bayesian data augmentation and the implementation of joint sampling of unobserved sequences and the transmission graph.

### The Stochastic Epidemic Process

We consider a broad class of spatio-temporal stochastic models exemplified by the SEIR epidemic model with susceptible (S), exposed (E), infectious (I) and removed (R) compartments. Suppose that we have a spatially distributed population indexed by 1, 2, …. Denote by *ξ*_*S*_(*t*), *ξ*_*E*_(*t*), *ξ*_*I*_(*t*) and *ξ*_*R*_(*t*) the set of indices of individuals who are in class S, E, I and class R respectively at time *t* and let *S*(*t*), *E*(*t*), *I*(*t*) and *R*(*t*) be the respective numbers in these classes at time *t*. An individual *j* ∈ *ξ*_*S*_(*t*) becomes exposed via primary infection with stochastic rate *α* and from an infection *i* ∈ *ξ*_*I*_(*t*) with rate *βK*(*d*_*ij*_;*κ*). The term *K*(*d*_*ij*_;*κ*) characterises the dependence of the infectious challenge from infective *i* to susceptible *j* as a function of distance between them *d*_*ij*_ and is known as the *spatial kernel function*[25, 27]. Here, we assume *K*(*d*_*ij*_;*κ*) = exp(−*κd*_*ij*_). Sources of infection are assumed to act independently of each other and combine so that the overall probability of *j* becoming infected during [*t*, *t* + *dt*) is given by

$$r\left(j,t,dt\right)=\left[\alpha +\beta \sum_{i\in \xi_{I}\left(t\right)}K\left(d_{ij};\kappa \right)\right]dt+o\left(dt\right).$$ (1)

We refer to *α* as the primary (background) transmission rate and *β* as the secondary transmission rate, and we note that the term *α* + *β*∑_*i* ∈ *ξ*_*I*_(*t*)_ *K*(*d*_*ij*_;*κ*) represents the total *hazard* of infection. Note that the magnitude of primary infection rate *α* is the determining factor for the number of primary cases and hence the number of clusters in the transmission graph.

Following exposure, the random times spent by individuals in classes *E* and *I* are modelled using an appropriate distribution such as a Gamma or a Weibull distribution [3, 4]. Specifically, we use a *Gamma*(*a*, *b*) parameterized by the shape *a* and scale *b* for the random time *x* spent in class *E* with density function $f_{E}\left(x;a,b\right)=\frac{1}{b^{a}\Gamma \left(a\right)}x^{a-1}e^{-\frac{x}{b}}.$ For the random time *x* spent in class *I* we use a *Weibull*(*γ*, *η*) parameterized by the shape *γ* and scale *η* with density function *f*_*I*_(*x*;*γ*, *η*) = (*η*/*γ*) (*x*/*γ*)^*η*−1^ *e* ^−(*x*/*γ*)^*η*^^. All sojourn times are assumed independent of each other given the model parameters. The various epidemic and ecological studies cited in the previous section make use of models that conform to this general framework.

### Stochastic Process for Genetic Evolution

The evolutionary process of the pathogen is modelled at the level of nucleotide substitutions. It is assumed that the nucleotide substitution process is independent over infected sites, conditional on the transmission graph and infection times. We assume that there is a single dominating strain/lineage at each infectious site at any time point (e.g. [16, 19, 20]) so that, upon exposure, the newly exposed individual is infected with this single dominant strain from the source individual. The dominant strain at an infected site evolves according to the continuous-time evolutionary process described below. Nucleotide bases at different positions of a sequence are assumed to evolve independently.

A nucleotide sequence is assembled from four nucleotide bases which can be classified into *purines* (e.g., adenine (*A*) and guanine (*G*) in both DNA and RNA viruses) and *pyrimidines* (i.e., thymine (*T*) and cytosine (*C*) in DNA viruses and uracil (*U*) and *C* in RNA viruses). Substitution between bases in the same category is called *transition* (not to be confused with the term *transition* in the context of a Markov process) and the substitution between bases from different categories is called *transversion*. Generally speaking, transversion occurs less frequently than transition. In keeping with common practice we model the mutation process by a continuous-time Markov process. Specifically we adopt the two-parameter *Kimura model* [28] (see also S1 Text *:A Markov Process to Model the Evolutionary Process*) which allows for different rates of transition and transversion. Taking RNA viruses as an example, we let *ω*_*N*_ = {*A*, *C*, *G*, *U*} be the set of nucleotide bases. Under the Kimura model, a nucleotide base *x* ∈ *ω*_*N*_ mutates to a nucleotide base *y* ∈ *ω*_*N*_ within an interval of arbitrary length △*t* with probability

$$P_{\mu_{1},\mu_{2}}\left(y|x,\Delta t\right)=0.25+0.25e^{-4\mu_{2}\Delta t}+0.5e^{-2(\mu_{1}+\mu_{2})\Delta t},\;\text{for}\;x=y\text{,}$$ (2a)

$$\begin{array}{l} P_{\mu_{1},\mu_{2}}(y|x,△t)\\ =\{\begin{array}{ll} 0.25+0.25e^{-4\mu_{2}△t}-0.5e^{-2(\mu_{1}+\mu_{2})△t}, & \text{for}\;x\neq y\;\text{specifying a transition},\\ 0.25-0.25e^{-4\mu_{2}△t}, & \text{for}\;x\neq y\;\text{specifying a transversion}, \end{array} \end{array}$$ (2b)

where *μ*_1_ and *μ*_2_ are the rates of transition and transversion respectively. Note that △*t* is arbitrary and does not have to be small for the equations above to hold. Moreover, this process is quite general and not restricted to modelling only RNA virus mutations.

### Multiple and Single Primary Infection Model

The assumption of having only one single primary infection during an outbreak has been shown to be applicable in many scenarios [19, 20]. This assumption has been more recently relaxed to allow for multiple initial infections – for example, [18] uses an *ad hoc* algorithm to detect genetic outliers and hence the imported cases, and [16] uses a sound post-processing algorithm to identify imported cases. To include multiple primary infections explicitly into our framework, we model the distribution of pathogen sequences from which the primary cases are drawn so that primary and secondary infections can be included and distinguished using the Bayesian computational procedures presented later.

*Background/primary sequences* (i.e. actual sequences passed to primary cases which initiated the clusters) are stochastic variants of a population characterised by a universal *master sequence*, *G*_*M*_, with each nucleotide base of the background/primary sequences sequence having a probability *p* (i.e. *variation parameter*) of differing from the base at the corresponding site in *G*_*M*_, in which case the base is drawn uniformly from the three possible alternatives. For example, if the *j* ^*th*^ position of the universal *master sequence* *G*_*M*_ is base *A*, the corresponding base passed to the *background/primary sequence* has probability $\frac{p}{3}$ of taking each of the values in the set *ω*_*N*_\*A* = {*C*, *G*, *U*} and has a probability 1 − *p* of being *A*. The completely drawn background/primary sequence may then evolve in time along the transmission in the initiated cluster. Also, deviations from *G*_*M*_ are assumed to be independent over sites. The universal master sequence (*G*_*M*_), the background/primary sequences that initiated clusters and the variation parameter (*p*) are all to be imputed (see later).

We note that, the background/primary sequences are largely constrained by the sampled sequences – an assumption made implicitly in [18] where genetic outliers are classified as imported cases. The universal master sequence *G*_*M*_ and the variation parameter *p* are considered as nuisance parameters, accommodating other scenarios concerning the process generating the background/primary sequences. For example, when two background/primary sequences that initiate two different clusters are actually derived from two distinct master sequences, the variation parameter *p* would be estimated to be large under the constraint of having only one master sequence. One may, of course, consider the two master sequences explicitly in the model. Nevertheless, we stress that the primary goal of having a primary infection model is to include more explicitly the primary sequences into our framework.

This multiple-cluster framework can be easily simplified to a single-cluster scenario considered in many practical problems (e.g. [19, 20]) by assuming that the initial exposure is drawn uniformly from all possible sites, that the sequence of the (initial) infecting strain drawn uniformly from all possible sequences, and that all subsequent exposures arise through secondary infection. Note that, in this case we are not required to represent explicitly the master sequence and the process generating the background/primary sequences.

### Complete-Data Likelihood

As the inferential procedures that we propose make extensive use of data augmentation we first discuss the formulation of a complete-data likelihood for the integrated epidemic/genetic model, bearing in mind that some of the quantities required to calculate the likelihood will be observed directly while others will be imputed.

Consider a population of *N* sites and assume that pathogen sequences comprise *n* bases. Suppose that we observe the epidemic between time *t* = 0 and *t* = *t*_*max*_, during which period the precise times and locations of all transitions between compartments are observed. Moreover, assume that for any exposure, the source of infection is also recorded, this being either primary infection or infection by a specific infectious host. Let *χ*_*S*_ denote the set of individuals remaining in class *S* at *t*_*max*_, and let *χ*_*E*_ ⊆ *χ*_*I*_ ⊆ *χ*_*R*_ denote the sets of individuals who have entered class *E*, class *I* and class *R* by *t*_*max*_ respectively. Also, let **E** = (…, *E*_*j*_, …) denote the exposure times for *j* ∈ *χ*_*E*_, **I** = (…, *I*_*j*_, …) denote the times of becoming infectious for *j* ∈ *χ*_*I*_ and **R** = (…, *R*_*j*_, …) denote the times of recovery or removal for *j* ∈ *χ*_*R*_. The cumulative distribution functions corresponding to the sojourn times in class *E* and class *I* are denoted by *F*_*E*_ and *F*_*I*_ respectively. Note that we use the term *exposure time* to denote the time of any transition from S to E, preferring not to use *infection time* in order to avoid potential confusion with times of transition from E to I.

Furthermore, to formulate the model it is necessary to allow recording of the sequences characterising the dominant pathogen strain at each exposed site *j* ∈ *χ*_*E*_ at potentially multiple times during the epidemic. Therefore, let *G*_⋅*j*_ = (*G*_1,*j*_, …, *G*_*m*_*j*_, *j*_) denote *m*_*j*_ sequences that characterise the dominant strain at site *j* ∈ *χ*_*E*_ at the corresponding (increasing) *sequencing times* *t*_⋅*j*_ = (*t*_1,*j*_, …, *t*_*m*_*j*_, *j*_). Note that *t*_⋅*j*_ includes the time of exposure for site *j*, *t*_1,*j*_ = *E*_*j*_ so that *G*_1,*j*_ characterises the strain transmitted to *j*. Also represented in *t*_⋅*j*_ are any times at which *j* passes infection to a susceptible host, so that strains transmitted from *j* are captured in *G*_⋅*j*_. Finally *t*_⋅*j*_ also includes the observed *sampling time* $t_{j}^{s}$ at which the dominant strain is sequenced at site *j*. We denote by ***G*** = (*G*_⋅1_, …, *G*_⋅*j*_, …) the complete set of nucleotide data formed. The transmission graph is specified by a vector *ψ* which records the source of infection *ψ*_*j*_ for each individual *j* ∈ *χ*_*E*_. Some key notation is summarised in Table 1.

**Table 1:** Key notation used in Models.

Notation	Description
*t*_⋅*j*_ = (*t*_1,*j*_, …, *t*_*m*_*j*_, *j*_)	The vector that contains *m*_*j*_ relevant *sequencing times* on exposed site *j* ∈ *χ*_*E*_.
*G*_⋅*j*_ = (*G*_1,*j*_, …, *G*_*m*_*j*_, *j*_)	The vector that contains corresponding *sequences* at times in the vector *t*_⋅*j*_.
$t_{j}^{s}$	The *observed sampling time* in the vector *t*_⋅*j*_.
$G_{1,j}^{k}$	The *nucleotide base* at *k* ^*th*^ position in the sequence *G*_1,*j*_.
*G*_*M*_ and $G_{M}^{k}$	The master sequence and its *k* ^*th*^-position nucleotide base.
*ψ*_*j*_	The source of infection for exposed site *j*.
*ω*_*N*_ = {*A*, *C*, *G*, *U*}	The set of nucleotide bases.
*ω*_*ψ*_ = {*i* ∈ *χ*_*I*_|*I*_*i*_ ≤ *t*_*u*_, *i* ≠ *ψ*_*j*_}	The set of candidates for a *new source of infection* for individual *j* with the current source of infection *ψ*_*j*_.

A sequence of events in which individual *i* infects individuals *j* and then *k* along with the sampling of sequences taken from these individuals is shown in Fig 1 to clarify the notation above. In practice, the observed data will only record the sampling times $t_{i}^{s}$, $t_{j}^{s}$, $t_{k}^{s}$ and the corresponding sequence samples (coloured grey) with all other quantities needing to be imputed. We will also consider the more general sampling situation where some exposures may never be sampled so that no sequence is recorded for them.

In the general multiple-cluster scenario, with complete data *z* = (**E**, **I**, **R**, **G**, **ψ**) and model parameters ***θ*** = (*α*, *β*, *a*, *b*, *γ*, *η*, *κ*, *μ*_1_,*μ*_2_, *p*), we can express the likelihood as

$$\begin{array}{cc} L(\theta ;z)= & \prod_{j\in \chi_{E}^{-1}}P\left(j,\psi_{j}\right)\times \exp\left\{-q_{j}\left(E_{j}\right)\right\}\times \prod_{j\in \chi_{S}}\exp\left\{-q_{j}\left(t_{max}\right)\right\}\\ & \times \prod_{j\in \chi_{I}}\;f_{E}\left(I_{j}-E_{j};a,b\right)\times \prod_{j\in \chi_{R}}\;f_{I}\left(R_{j}-I_{j};\gamma ,\eta \right)\\ & \times \prod_{j\in \chi_{E\backslash I}}\left\{1-F_{E}\left(t_{max}-E_{j};a,b\right)\right\}\times \prod_{j\in \chi_{I\backslash R}}\left\{1-F_{I}\left(t_{max}-I_{j};\gamma ,\eta \right)\right\}\\ & \times \prod_{j\in \chi_{E}}\;g\left(G_{2,j},\ldots ,G_{m_{j},j}|t_{\cdot j},\psi_{j},G_{1,j}\right)\times \prod_{j\in \chi_{E}}h\left(G_{1,j}|\psi_{j}\right). \end{array}$$ (3)

Here $\chi_{E}^{-1}$ denotes *χ*_*E*_ with the earliest exposure (which must be a primary infection) excluded. The contribution to the likelihood arising from the infection of *j* by the particular source *ψ*_*j*_ is given by

$$\begin{array}{c} P\left(j,\psi_{j}\right)=\left\{\begin{array}{cc} \alpha , & \text{if}\;\text{individual}\;j\;\text{is}\;\text{a}\;\text{primary}\;\text{case,}\\ \beta K(d_{\psi_{j}j};\kappa ), & \text{if}\;\psi_{j}\;\in \chi_{I}\;\text{at}\;\text{time}\;E_{j}\text{.} \end{array}\right. \end{array}$$ (4)

We define

$$q_{j}\left(s\right)=\int_{0}^{s}\left\{\alpha +\sum_{i\in \xi_{I}\left(t\right)}\beta K\left(d_{ij};\kappa \right)\right\}dt,$$ (5)

so that the terms exp{−*q*_*j*_(*E*_*j*_)} and exp{−*q*_*j*_(*t*_*max*_)} give the contribution to the likelihood arising from the survival of each exposed individual until its respective exposure time or, in the case of non-exposed individuals, until *t*_*max*_. The second and third lines in Eq 3 represent the contribution to the likelihood of the sojourn times in class E and I respectively.

Terms in the last line in Eq 3 carry the contribution to the complete-data likelihood of the sequence data. The term

$$\begin{array}{c} g\left(G_{2,j},\ldots ,G_{m_{j},j}|t_{\cdot j},\psi_{j},G_{1,j}\right)=\prod_{i=1}^{n}\prod_{k=1}^{m_{j}-1}P_{\mu_{1},\mu_{2}}\left(G_{k+1,j}^{i}|G_{k,j}^{i},\Delta t=t_{k+1,j}-t_{k,j}\right) \end{array}$$ (6)

gives the probability that, conditional on the infecting strain (i.e., *G*_1,*j*_) and the sampling times, a given sequence of mutations (to be inferred) occurs in the exposed individual *j*. The term *p*_*μ*_1_,*μ*_2__(⋅) is defined in Equation 2 (where $G_{k,j}^{i}$ denotes the nucleotide base at position *i* of sequence *k* on individual *j*).

The expression *h*(*G*_1,*j*_|*ψ*_*j*_) represents the contribution to the likelihood arising from the infecting strain, and is given by

$$\begin{array}{c} h\left(G_{1,j}|\psi_{j}\right)=\left\{\begin{array}{cc} {\left(\frac{p}{3}\right)}^{l_{j}}{\left(1-p\right)}^{n-l_{j}}, & \text{if}\;\text{individual}\;j\;\text{is}\;\text{a}\;\text{primary}\;\text{case,}\\ 1, & \text{if}\;\psi_{j}\;\in \chi_{I}\text{,} \end{array}\right. \end{array}$$ (7)

where *p* (the variation parameter) is the probability that a base of *G*_1,*j*_ is different from the base at the corresponding position of the given *master sequence* *G*_*M*_ and *l*_*j*_ is the total number of differing bases. The term $\frac{1}{3}$ reflects the assumption that a base is randomly chosen from a uniform distribution on the set $\omega_{N}\backslash G_{M}^{i}$, where $G_{M}^{i}$ is the nucleotide base on *i* ^*th*^ position of the master sequence.

The likelihood for the single-cluster scenario is obtained simply by discarding the factor ∏_*j* ∈ *χ*_*E*__ *h*(*G*_1,*j*_|*ψ*_*j*_).

### A Systematic Bayesian Integration Framework

It is now standard practice to conduct Bayesian analyses of partially observed epidemics using the process of *data augmentation* supported by computational techniques such as Markov chain Monte Carlo methods [1, 3, 25, 29]. Given observed partial data ***y***, such as times of symptom onset or culling times, these approaches involve sampling from the joint posterior distribution *π*(***θ***, ***z***|***y***) ∝ *L*(***θ***;***z***)*π*(***θ***), where ***z*** represents the complete data and *π*(***θ***) represents the prior distribution of model quantities, such that the complete ***z*** is reconstructed, or ‘imputed’. In our application, ***z*** involves both partially observed epidemic and sequence data.

As discussed in *Introduction*, a crucial research challenge for the joint inference of epidemic and molecular evolution processes is to devise a statistically sound, and computationally efficient algorithm for the joint imputation of the unobserved sequences, the transmission graph ***ψ*** and the unobserved infection times ***E***. In this section we describe how the unobserved ***ψ*** and the unobserved sequences in ***G*** may be updated along with the unobserved exposure times ***E***, this being the key challenge in devising a suitable algorithm. The analysis takes about 2 to 17 hours to run on a single-core computer, depending on the amount of genomic data used (see details in S1 Text *:Computing Time and Other Benchmarks*). Details of more standard elements of the MCMC algorithm are also described in S1 Text *:Supplementary Details of the MCMC Algorithm*. Beside using extensive simulations, our methods have also been tested and validated by mathematical arguments and specifically-designed computer experiments (for details see S1 Text *:Validation of the Methodology*).

#### Sampling from the posterior: and overview of the MCMC algorithm

Given the complexity of the model and data structure (and hence of the notation) being considered in this paper, we first give an overview of the key elements of the algorithm before presenting their precise mathematical descriptions. **Part I** of the algorithm allows us to sample jointly the exposure time and the corresponding sequences transmitted from the donor to the recipient (without changing the source of infection). The basic idea is to propose a new sequence somewhere between two “known” sequences at either side of a newly proposed exposure time, where a “known” sequence can either be an observed or imputed sequence. The source of infection is sampled in **Part II** of the algorithm, jointly with the exposure time and the transmitted sequence − a new source of infection for an individual *j* is randomly chosen among all infectious sites according to the infectious challenges presented to *j*; conditioning on the sampled new source of infection, a new exposure time and transmitted sequence are proposed in a similar way to Part I. By sequentially applying this algorithm to all exposures, the complete set of transmitted sequence in ***G***, the transmission graph ***ψ*** and the exposure times ***E*** are updated. To further facilitate reading of the current and following sections some key notation is summarised in Table 1.

#### Part I: Joint sampling of the exposure time *E* and the unobserved sequences in *G*

Assuming for now that the source of infection *ψ*_*j*_ is unchanged, and given the current exposure time *E*_*j*_ for individual *j* and the corresponding sequence *G*_1,*j*_, we first propose a new exposure time $E_{j}^{{}^{\prime }}$ using a standard approach (see S1 Text *:Supplementary Details of the MCMC Algorithm* for details). Here we describe in detail how a suitable candidate for the corresponding sequence *G*′_1,*j*_ can be simultaneously proposed.

**Fig 2:**
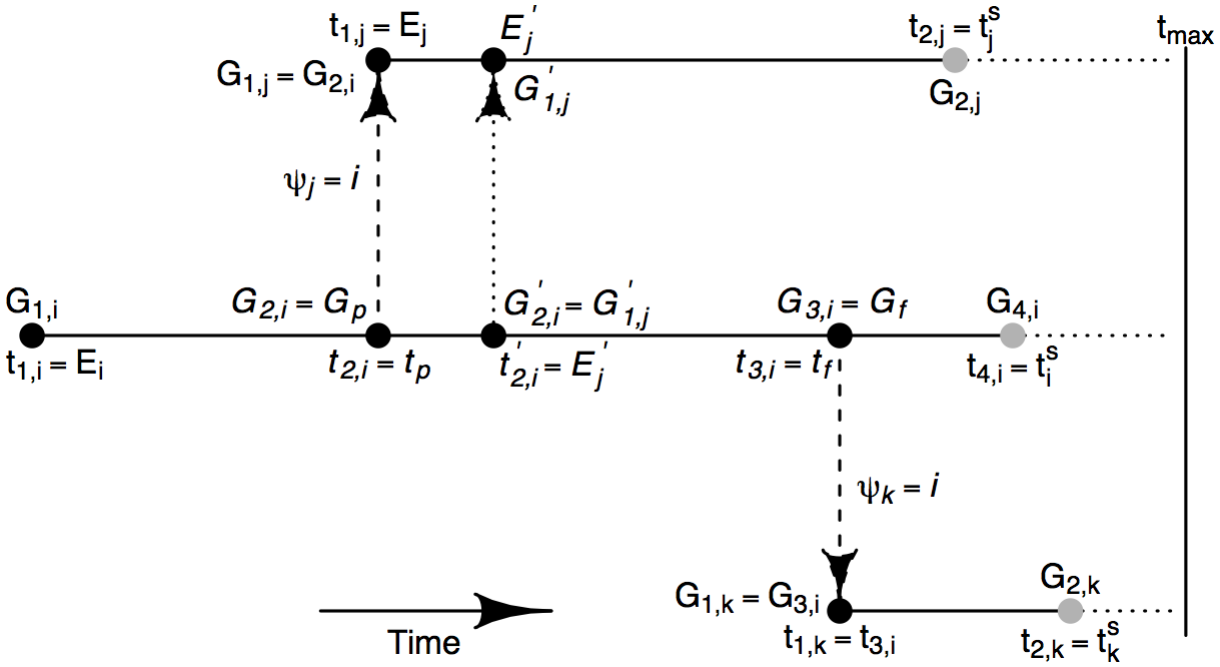
Illustration of the selection *t*_**p**_ (and the corresponding past sequence *G*_**p**_) and *t*_**f**_ (and the corresponding past sequence *G*_**f**_) (see also main text).

The key idea is to propose a new sequence at $E_{j}^{{}^{\prime }}$ which has plausible proximity to a *nearest* **past** *sequence* *G*_**p**_ and a *nearest* **future** *sequence* *G*_**f**_ relative to $E_{j}^{{}^{\prime }}$. Throughout *past* and *future* are defined with respect to the direction of $\Delta E_{j}=E_{j}^{{}^{\prime }}-E_{j}$. Therefore, if $E_{j}^{{}^{\prime }}$ precedes *E*_*j*_ then *G*_**p**_ corresponds to a later (absolute) time than *G*_**f**_. We choose *G*_**p**_ and *G*_**f**_ by taking account of the sequences both from individual *j* and the source of infection *ψ*_*j*_, to which no change is proposed in this operation. Denoting *t*_**p**_ and *t*_**f**_ as the sequencing times for *G*_**p**_ and *G*_**f**_ respectively, we have

$$t_{p}=\min_{\left|t-E_{j}^{{}^{\prime }}\right|}\left\{t\in t_{\cdot j}\cup t_{\cdot \psi_{j}}|\mathrm{sgn}\left(t-E_{j}^{{}^{\prime }}\right)\neq \mathrm{sgn}\left(\Delta E_{j}\right)\right\}$$ (8)

and

$$t_{f}=\min_{\left|t-E_{j}^{{}^{\prime }}\right|}\left\{t\in t_{\cdot j}\cup t_{\cdot \psi_{j}}|\mathrm{sgn}\left(t-E_{j}^{{}^{\prime }}\right)=\mathrm{sgn}\left(\Delta E_{j}\right)\right\},$$ (9)

where *sgn* is the *signum* *function* (see S1 Text).*G*_**p**_ (or *G*_**f**_) is taken to be the corresponding sequence on individual *j* whenever *t*_**p**_ (or *t*_**f**_) is represented in both *t*_⋅*j*_ and *t*_⋅*ψ*_*j*__. This is illustrated in Fig 2 where a new exposure time $E_{j}^{{}^{\prime }}$ for individual *j* from Fig 1 is proposed. Here *t*_**p**_ and *t*_**f**_ are taken to be the current exposure time *E*_*j*_ and *t*_3,*i*_ respectively. Then, by definition, the corresponding sequences at *t*_**p**_ and *t*_**f**_ are *G*_**p**_ = *G*_1,*j*_ and *G*_**f**_ = *G*_3,*i*_ respectively. Note that *G*_2, *i*_ and *t*_2, *i*_ are also simultaneously updated.

Given the nucleotide base $G_{p}^{k}$ and $G_{f}^{k}$ at the *k* ^*th*^ position and **Δ** *t*_**p**_ = |*t*_**f**_ − *t*_**p**_|, by conditioning on at most one change occurring at each position in the sequence during the period **Δ** *t*_**p**_, and assuming a linear relationship between the probability of change and the time duration, we have

$$\begin{array}{c} G_{1,j}^{{}^{\prime }k}=\left\{\begin{array}{cc} G_{f}^{k}, & \text{with}\;\text{probability}\;P_{f}=\frac{\left|E_{j}^{{}^{\prime }}-t_{p}\right|}{\Delta t_{p}}\text{,}\\ G_{p}^{k}, & \text{with}\;\text{probability}\;1-P_{f}\text{.} \end{array}\right. \end{array}$$ (10)

As **Δ** *t*_**p**_ is generally small, we allow only one change during the time interval for a particular nucleotide base, which is not entirely consistent with the assumption of a continuous-time Markov process. In order to explore thoroughly the domain of ***G***, ***G*** is also updated independently of the exposure times (see S1 Text *:Supplementary Details of the MCMC Algorithm*).

It is noted that *t*_**p**_ and *G*_**p**_ are always well-defined as the corresponding set in Eq 8 is always non-empty and contains *E*_*j*_. On the other hand *t*_**f**_ and *G*_**f**_ may be undefined as the corresponding set in Eq 9 can be empty. If *G*_**f**_ is not well-defined, we propose $G_{1,j}^{{}^{\prime }}$ according to the mechanism defined in Equation 2 such that, for each *k* independently, a move from $G_{p}^{k}$ to $G_{1,j}^{{}^{\prime }k}=y$ is proposed with probability

$$P\left(G_{1,j}^{{}^{\prime }k}=y|G_{p}^{k},\Delta t=\left|E_{j}^{{}^{\prime }}-t_{p}\right|\right)=p_{\mu_{1},\mu_{2}}\left(G_{1,j}^{{}^{\prime }k}=y|G_{p}^{k},\Delta t=\left|E_{j}^{{}^{\prime }}-t_{p}\right|\right).$$ (11)

When *ψ*_*j*_ ∉ *χ*_*I*_ (i.e., *j* is a primary infection), and when *G*_2, *j*_ is not available, the newly proposed sequence is not constrained to match any other sequence. In this situation the proposal distribution simply reflects the assumptions regarding the background sequence. Specifically, $G_{1,j}^{{}^{\prime }k}$ has a probability 1 − *p* of matching the corresponding site $G_{M}^{k}$ in the master sequence *G*_*M*_. Otherwise the base is randomly drawn from the set $\omega_{N}\backslash G_{M}^{k}$.

Lastly, the proposed update of the current data ***z*** to ***z***′ is accepted with a M-H acceptance probability (see S1 Text *Supplementary Details of the MCMC Algorithm*). By sequentially applying this algorithm to all exposures *j* ∈ *χ*_*E*_, ***E*** and ***G*** can be jointly updated.

#### Part II: Joint sampling of the transmission graph *ψ*, the exposure time *E* and the unobserved sequences in *G*

Denote *t*_*u*_ as the upper limit of $E_{j}^{{}^{\prime }}$ (see S1 Text *:Supplementary Details of the MCMC Algorithm*) and *ω*_*ψ*_ = {*i* ∈ *χ*_*I*_|*I*_*i*_ ≤ *t*_*u*_, *i* ≠ *ψ*_*j*_} as the set of candidates for a new source of infection $\psi_{j}^{{}^{\prime }}$. We propose a new infecting source *i* ∈ *ω*_*ψ*_ to be $\psi_{j}^{{}^{\prime }}$ with probability

$$s_{ij}\propto \beta K\left(d_{ij};\kappa \right).$$ (12)

Note that, for the multiple-cluster scenario, the primary infection can be accommodated by adding a permanent infectious source presenting an additional challenge of strength *α* to individual *j*. Having proposed $\psi_{j}^{{}^{\prime }}$, $E_{j}^{{}^{\prime }}$ can subsequently be proposed (see S1 Text *:Supplementary Details of the MCMC Algorithm*) with consequent proposed changes to $t_{\cdot j}^{{}^{\prime }}$ and $t_{\cdot \psi_{j}^{{}^{\prime }}}^{{}^{\prime }}$.

The proposal of the new sequence $G_{1,j}^{{}^{\prime }}$ differs from last section as *E*_*j*_ and *G*_1,*j*_ become irrelevant when the source of infection also changes. In the case of a new source $\psi_{j}^{{}^{\prime }}\in \chi_{I}$ we define

$$t_{p}=\min_{\left|t-E_{j}^{{}^{\prime }}\right|}\left\{t\in t_{\cdot \psi_{j}^{{}^{\prime }}}^{{}^{\prime }}|t<E_{j}^{{}^{\prime }}\right\},$$ (13)

where $t_{\cdot \psi_{j}^{{}^{\prime }}}^{{}^{\prime }}$ indicates the updated sequencing times on $\psi_{j}^{{}^{\prime }}$ (which is simultaneously updated after the updates of *E*_*j*_ and *ψ*_*j*_) and then we can identify the respective sequence *G*_**p**_. Also, we define

$$t_{f}=\min_{\left|t-E_{j}^{{}^{\prime }}\right|}\left\{t\in t_{\cdot j}^{{}^{\prime }}\cup t_{\cdot \psi_{j}^{{}^{\prime }}}^{{}^{\prime }}|t>E_{j}^{{}^{\prime }}\right\},$$ (14)

where $t_{\cdot j}^{{}^{\prime }}$ indicates the updated sequencing times on *j*. Note that *t*_**f**_ > *t*_**p**_ always holds in the definitions in this case. *G*_**f**_ is taken to be the corresponding sequence on individual *j* whenever *t*_**f**_ is in both $t_{\cdot j}^{{}^{\prime }}$ and $t_{\cdot \psi_{j}^{{}^{\prime }}}^{{}^{\prime }}$. $G_{1,j}^{{}^{\prime }}$ is then sampled according to Eq 10. Similarly, $G_{1,j}^{{}^{\prime }}$ is sampled according to Eq 11 when *G*_**f**_ is not well-defined.

In the case of $\psi_{j}^{{}^{\prime }}\notin \chi_{I}$, we let *G*_**p**_ = *G*_2, *j*_ and sample $G_{1,j}^{{}^{\prime }}$ according to Eq 11; if *G*_2, *j*_ is not available, $G_{1,j}^{{}^{\prime }i}$ is drawn from the distribution of the background sequences described in last section. Once the new source, sequence and exposure time are proposed, the proposed update from ***z*** to ***z***′ is accepted with a M-H acceptance probability (see S1 Text *:Supplementary Details of the MCMC Algorithm*). Similar to the last section, updates are sequentially applied to all exposures *j* ∈ *χ*_*E*_ so that ***ψ*** and ***E*** and ***G*** can be jointly updated.

## Results

### Simulation Studies

#### Valuation of genetic data

In this section we perform inference of transmission dynamics based on epidemics simulated under conditions that reflect real-world scenarios, with the primary aim of assessing the performance of our inference framework in a range of circumstances. We also characterise and quantify systematically the importance of genetic data for inference of a few important aspects of epidemic dynamics: the transmission graph, epidemiological parameters and the assignment of infections to the clusters. Moreover we demonstrate that genetic data may also facilitate model assessment using methods recently developed by the authors [27].

Specifically, we investigate the effect of having partial genetic data in two different ways that bring insights for the design of future studies:

1. Similar to [16], we investigate the effect of *sub-sampling of exposures* which allows that sequence samples may be available for only a subset of exposures.
2. Motivated by economic and computational (time) considerations, we investigate the effect of *partial genome sequencing* whereby a reduced number of bases are recorded in the sequences collected.

Note that as the transmitted sequences are imputed in our algorithm, unsampled exposures (i.e. infected hosts without observed sequence samples) can be naturally accommodated and their effect can be therefore studied.

In studying the effect of *sub-sampling of exposures*, we consider scenarios where a sequence sample and the corresponding sampling time may have a fixed exclusion probability from the observed data. To facilitate comparison, any scenario with a higher sampling percentage includes observed samples from all scenarios with lower sampling percentages. Also note that when no genetic data are available (i.e., 0% of the exposures are sampled) only the epidemic model described in section *Model and Methods* is fitted.

#### Simulated epidemics with multiple clusters

To test our algorithm we first apply it to analyse spatio-temporal, multiple-cluster epidemics simulated in a population of size *N* = 150 (comparable to those found in practical applications [16, 18, 20]). Their locations are generated independently from a uniform distribution over a square region, between times *t* = 0 and *t* = *t*_*max*_ = 60 (days). We choose model parameters for simulated scenarios using values arising from practical considerations [12, 19, 20, 27]. We assume that the epidemic begins at time 0 and evolves according to Eq (1). We initially set *α* = 0.0004, *β* = 8.0, *K*(*d*_*ij*_, *κ*) = exp(−0.02*d*_*ij*_), and assume that the sojourn times in classes E and I follow *Gamma*(10, 0.5) and *Weibull*(2, 2) distributions respectively. Pathogen sequences of length *n* = 8000 are transmitted upon infection and evolve according to Equation (2) with *μ*_1_ = 0.002 (bases per day) and *μ*_2_ = 0.0005 (bases per day). Each base of the master sequence *G*_*M*_ is drawn uniformly from the set *ω*_*N*_ = {*A*, *C*, *G*, *U*} and we let *p* = 0.01. We also perform simulations with a higher primary transmission rate (with a correspondingly larger expected number of clusters) and using higher mutation rates. For this second scenario, we have *α* = 0.002, *β* = 8.0, *μ*_1_ = 0.003, *μ*_2_ = 0.001 with other model parameters the same as those used above.

Exemplar simulations with these two sets of parameters give rise to a 3-cluster epidemic (147 out of 150 farms are infected) and a 6-cluster epidemic (all 150 farms are infected) respectively. The observations ***y*** consist only of the observed sequences sampled from exposed individuals and the corresponding known sampling times, a bounded range of the times and the precise locations of transitions from E to I (see also S1 Text *:Supplementary Details of the MCMC Algorithm*), and the precise times and locations of transitions from I to R that occur during the observation period.

We demonstrate the feasibility of imputing the distribution of background sequences and hence allow inference of multiple-cluster transmission graphs. Specifically, we impute the master sequence *G*_*M*_ (see S1 Text *:Supplementary Details of the MCMC Algorithm*) and the model parameter *p* along with the imputations of other model parameters and unobserved data.

#### Estimating the transmission graph and other epidemiological-evolutionary dynamics

**Fig 3:**
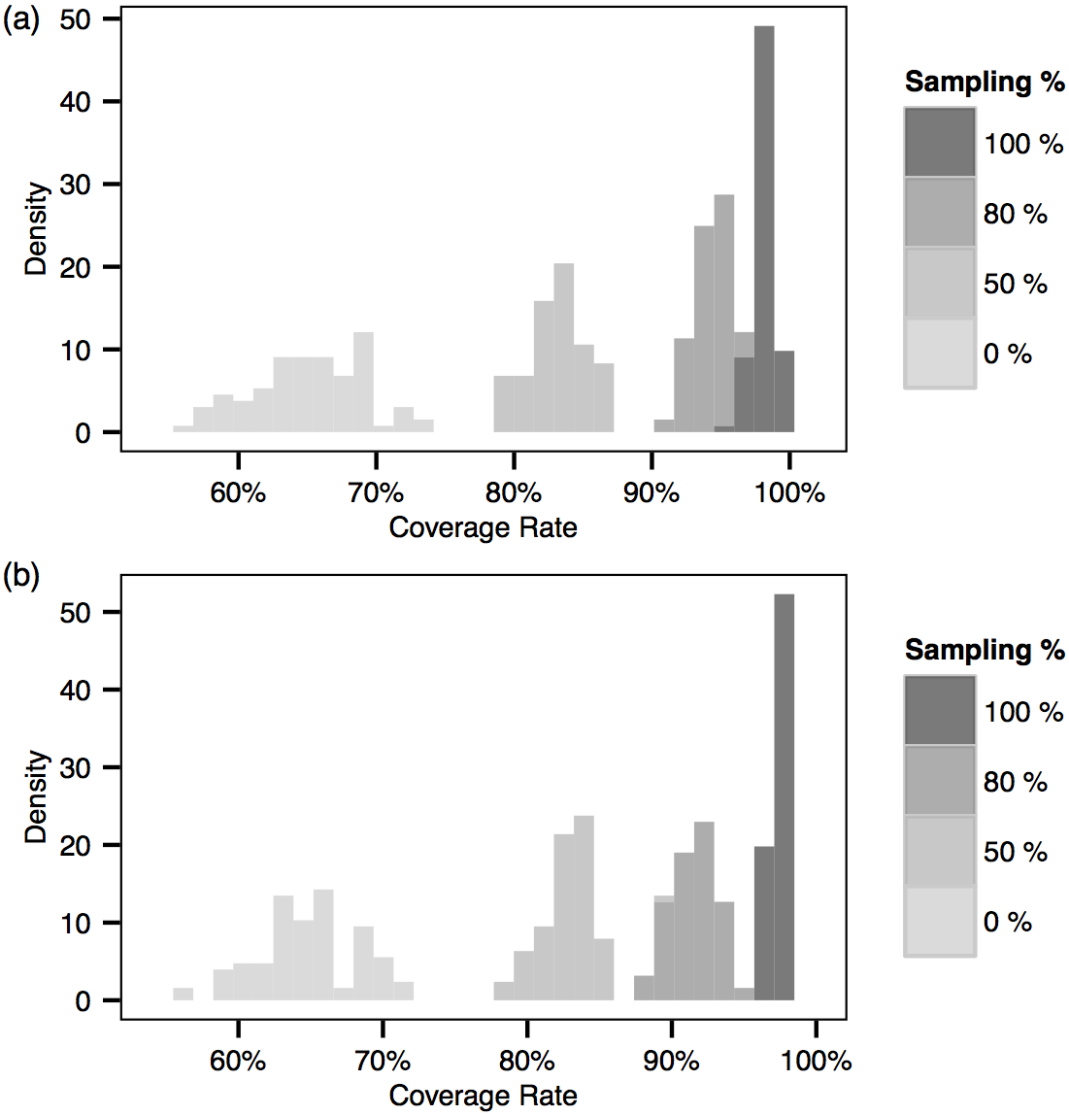
Posterior distributions of the overall coverage rate for the two multiple-cluster epidemics. (a) 3-cluster. (b) 6-cluster.

The (overall) *coverage rate* of an imputed transmission graph is defined as the proportion of infections for which the correct source is identified in the network. The posterior distribution of the coverage rate is therefore a useful indicator of how well the imputed networks match the true network. From Fig 3 we first notice that in the case with full sampling the transmission graph is typically recovered with near-complete accuracy. It is clear that the mean of the posterior distribution of the coverage rate increases with the proportion of exposed individuals being sampled.

Note that we have considered scenarios with relatively rich epidemiological data. In particular, we have considered data scenarios where the times of becoming infectious are known within a range or window and where the recovery times are observed (see also S1 Text *:Supplementary Details of the MCMC Algorithm*). In practice, particularly for animal disease outbreaks, they may be typically inferred from symptoms onset data and culling times [12, 20]. Although in the scenarios considered here the transmission graph may still be estimated with certain accuracy without genetic data, we observe a significantly larger variance in the absence of genetic data, and the added accuracy (both in terms of mean and variance) gained from genetic information is clear. Note that our estimation of the benefit of genetic data is likely to be conservative; in scenarios where the epidemiological data is less rich the value of genetic information is likely to be even greater that than that shown in this paper.

**Fig 4:**
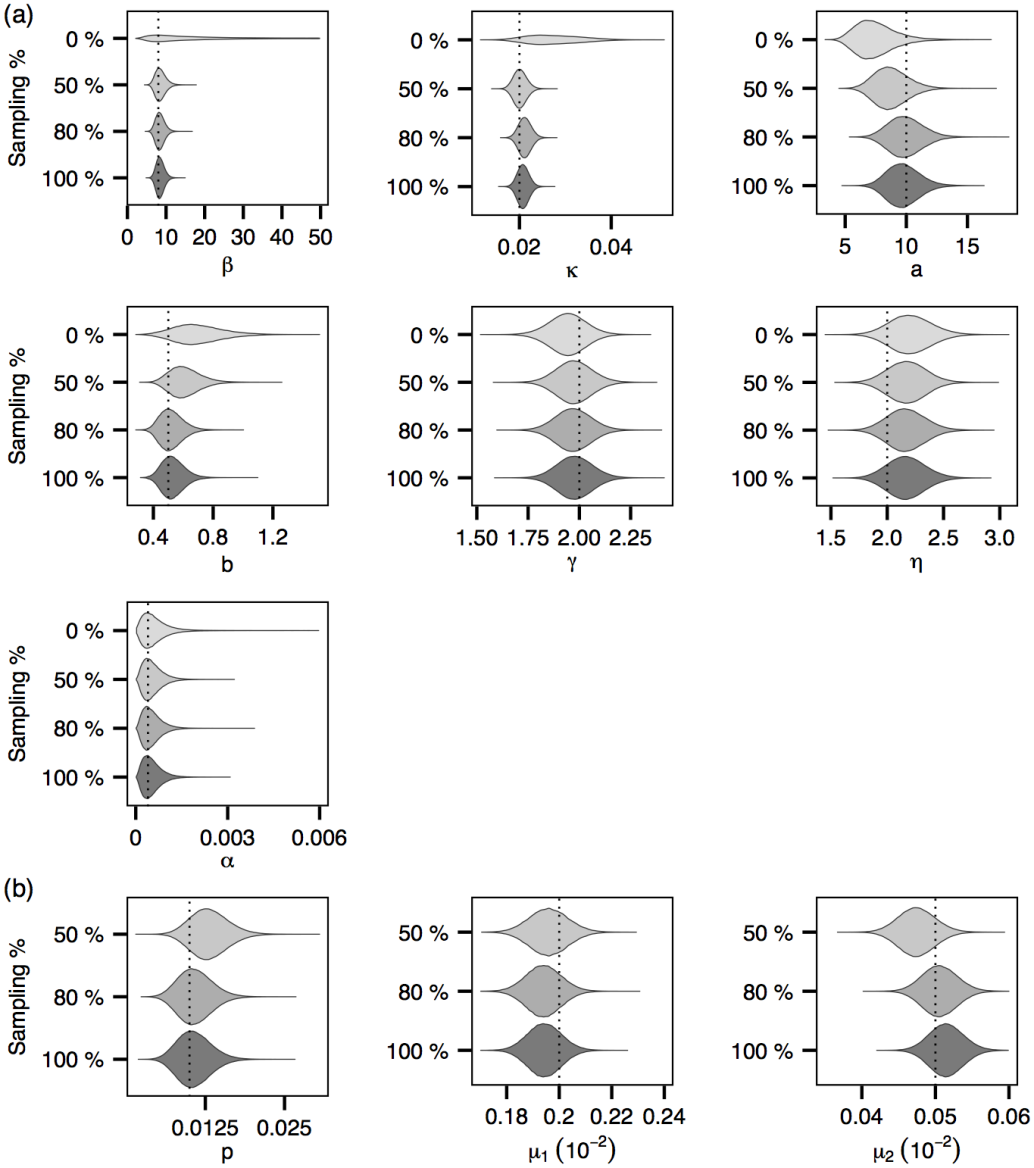
Posterior distributions of the model parameters (with the *3-cluster* epidemic). Dotted lines represent the true values of the model parameters. (a) Epidemiological parameters. (b) Evolutionary model parameters.

**Fig 5:**
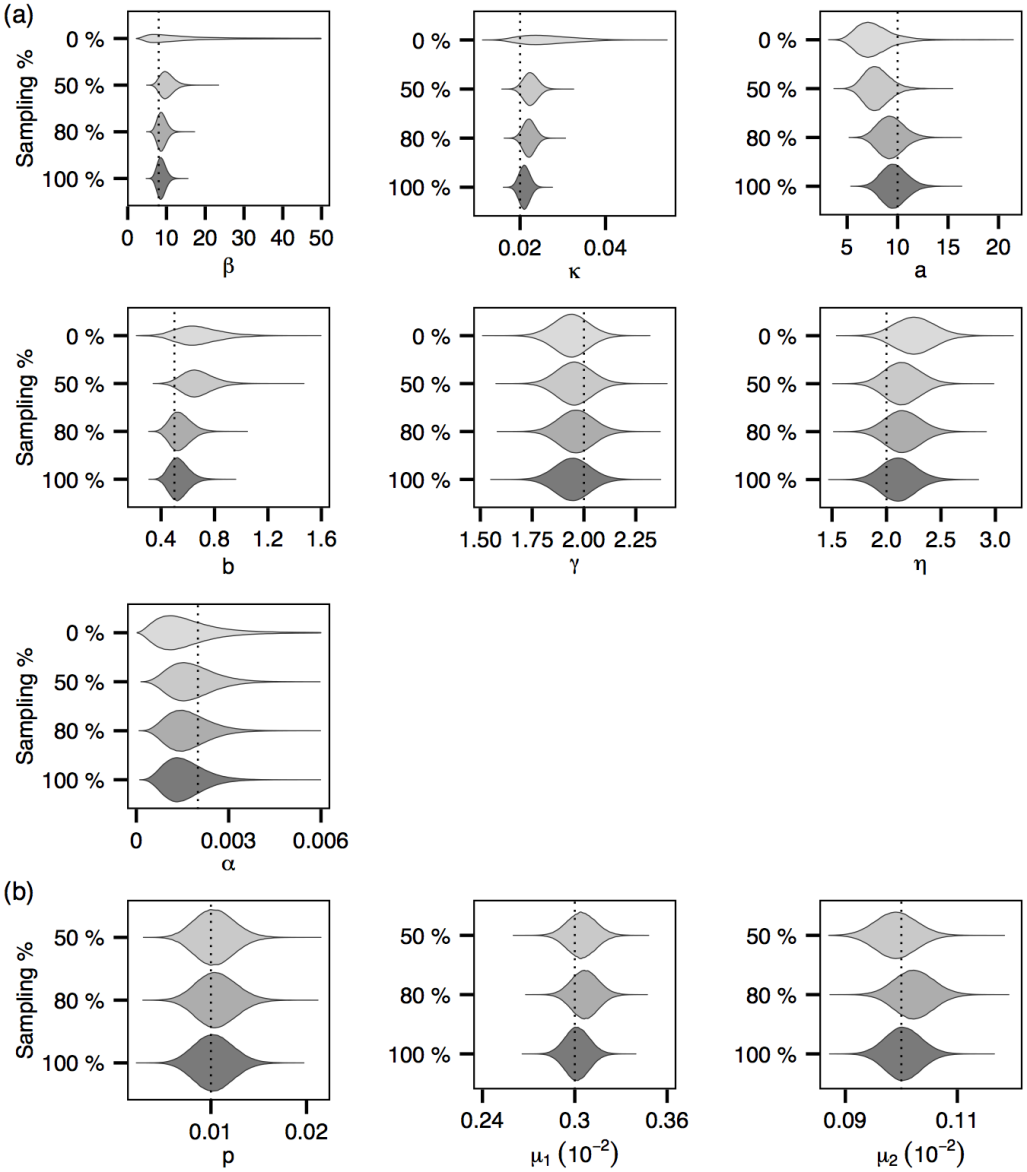
Posterior distributions of the model parameters (with the *6-cluster* epidemic). (a) Epidemiological parameters. (b) Evolutionary model parameters.

Figs 4 and 5 show the posterior distributions of the model parameters corresponding to three- and 6-cluster epidemics respectively. Figs 4(a) and 5(a) show that in general the credible intervals of the epidemiological parameters become narrower as more genetic data become available. This trend appears to be most prominent for *β* and *κ*, which is not surprising given their roles in determining the transmission graph and the fact that, as shown in Fig 3, the transmission graph is more accurately estimated when genetic data are more readily available. Figs 4(b) and 5(b) show similar but much less prominent trends for the genetic model parameters. Note that, as the times of transitions from E to I are known within a bounded range (see also S1 Text *:Supplementary Details of the MCMC Algorithm*), we do not observe significant differences among the scenarios for parameters *γ* and *η*. When the proportion of sampling further reduces, the estimates of model parameters, especially for the mutation rates and model parameters of latent period distributions, become less robust and we are not able to obtain reliable estimates systematically (i.e. the Markov chains often do not converge).

#### Estimating the number of clusters

Table 2 shows that the number of clusters, *N*_*c*_, is well-recovered by the posterior samples with a slight tendency towards over-estimation when the proportion of sampling reduces. Note that, also, the variances of *N*_*c*_ in the scenarios without genetic data are significantly larger. We also present the posterior distributions of the imputed master sequences in Table S4 to S6 in S1 Text.

**Table 2:** Summaries of the posterior distribution of the number of cluster *N*_*c*_. The mean of number of clusters is followed by the standard deviation in brackets.

Sampling%	100%	80%	50%	0%
*N*_*c*_ (3-cluster)	3.04 (0.21)	3.08 (0.27)	3.13 (0.39)	3.73 (2.84)
*N*_*c*_ (6-cluster)	6.0 (0.0)	6.50 (0.70)	6.91 (1.02)	6.75 (5.06)

#### Identifying the sources of clusters

**Fig 6:**
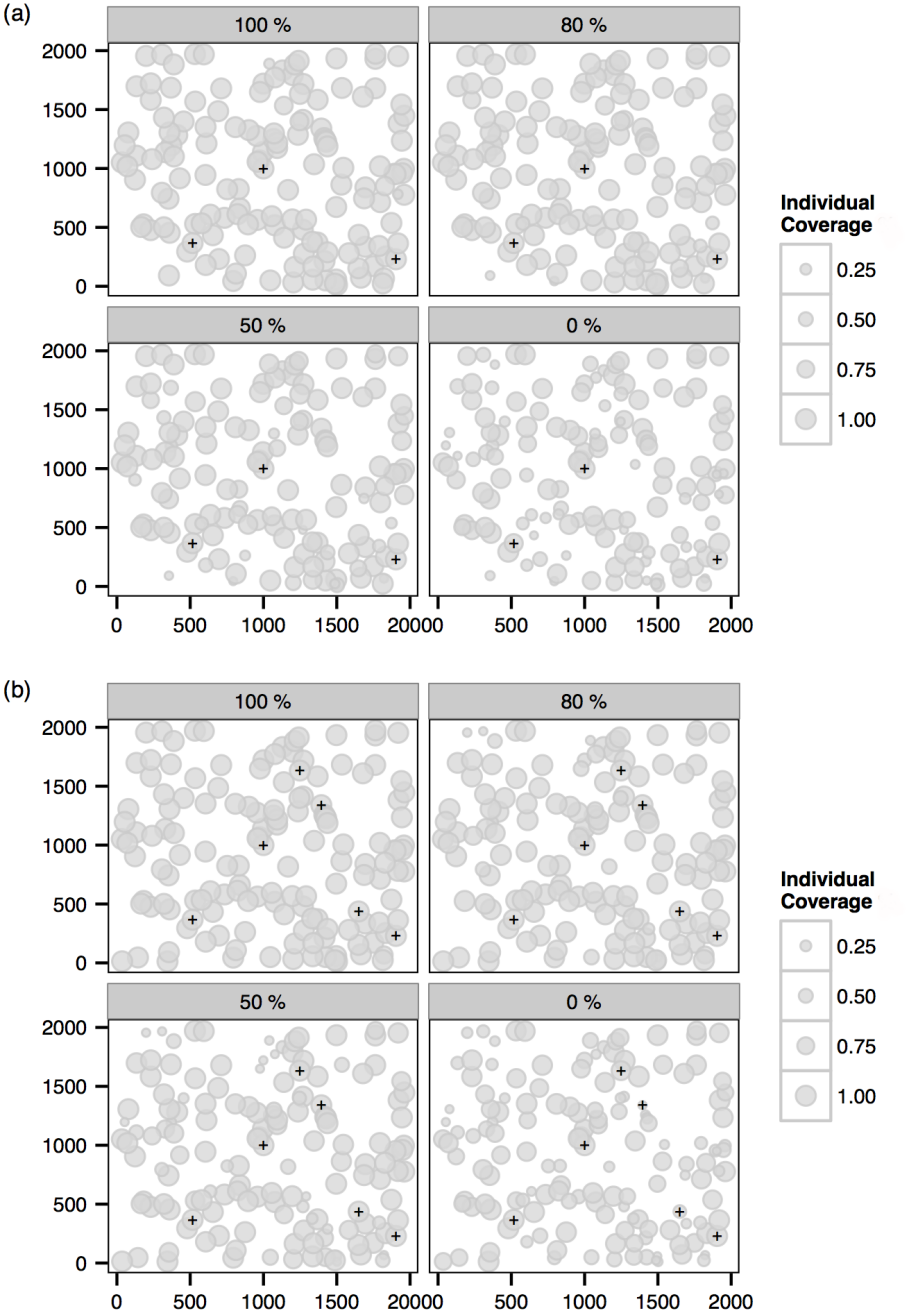
Posterior *individual coverage* of sources of infection (see main text) in scenarios with sampling 100%, 80%, 50% and 0%. The size of bubbles represent the coverage rate for a particular case at the corresponding position. The black + indicate the actual primary cases. (a) 3-cluster. (b) 6-cluster. Note that epidemics are simulated within a continuous 2000×2000 square region.

The (overall) coverage rate gives a broad measure of the recovery of the transmission graph. Here we examine the posterior distribution of the source of infection of a particular exposure. Define the posterior *individual* coverage rate for a particular infection to be the proportion under the posterior distribution of the transmission graph with which the true source of infection is correctly identified. Fig 6 shows the posterior individual coverage rate of all exposures at scenarios with different sampling percentages. We note that the individual coverage rate in general increases with the sampling percentage. It is also apparent that the primary infections (indicated by the symbol +) are frequently correctly identified (evidenced by high individual coverage rates), particularly in the scenarios with sequence samples.

Another natural question to ask is whether identification of the clusters of transmission, which helps identification of risk factors and yields useful insights into devising effective control strategies [30, 31], can be achieved accurately by our analysis. In order to investigate this we consider two measures that can be calculated over posterior samples of the transmission graph and whose posterior expectations quantify the accuracy with which clusters arising from a given primary infection are identified in the inference. These are as follows.

1. For each infection we estimate the *cluster identification rate*, this being the proportion under the posterior distribution of the transmission graph with which the true primary infection leading to the given infection is correctly identified (i.e., the correct primary infection appears *as the root* of the sub-graph containing the given infection).
2. For each infection we estimate the *(primary) ancestor identification rate*, namely the proportion under the posterior distribution of transmission graph with which the true primary infection leading to the given infection appears *on the path* from the infection to the root of the sub-graph.

Clearly, measure (1) will be lower than (2) since the conditions for ‘success’ are stronger. By estimating these quantities, we are able to quantify the extent to which the link between primary and secondary infections, and hence the clusters of transmission, is accurately identified in the inferential procedure. For a given transmission graph, we can identify the total number of infections that are linked to the correct primary infection according to the criteria used in the definition of (1) and (2) above to provide two alternative summary statistics of the graph that capture the extent to which attribution to primary infection has been inferred in the graph.

**Fig 7:**
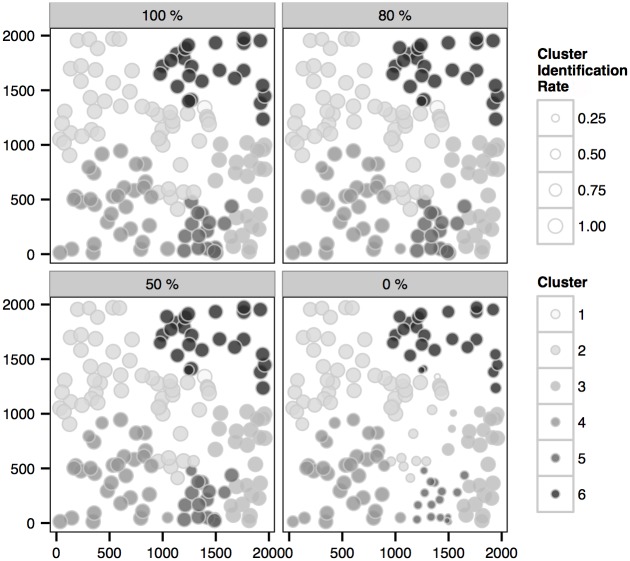
Posterior *cluster identification rate* of the infections (see main text), within each actual cluster of the *6-cluster* epidemic, in scenarios with sampling 100%, 80%, 50% and 0%.

**Fig 8:**
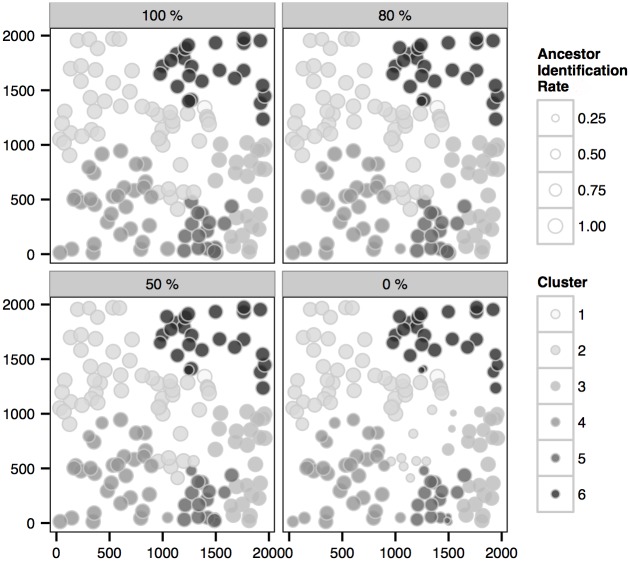
Posterior (primary) *ancestor identification rate* of the infections (see main text), within each actual cluster of the *6-cluster* epidemic, in scenarios with sampling 100%, 80%, 50% and 0%.

Here we focus on the analysis of the 6-cluster epidemic. From Figs 7 and 8 we first notice that the primary-to-secondary infection links, and hence the clusters, can be reasonably inferred in the scenarios with sequence samples. Also, the difference between high and low sampling levels is insignificant compared to the difference of individual coverage rates observed in Fig 6(b) and to the difference of overall coverage rates observed in Fig 3. These results indicate that the clusters may be accurately identified even in scenarios with a relatively small percentage of sampling while the transmission graph may be less accurately inferred. Note that in the scenario with no sequence data the cluster identification rate for cluster 5 is low (see Fig 7), which indicates that the root of the cluster is not frequently identified as a primary infection (see also Fig 6(b)); nevertheless, the ancestors of the cases in this cluster can be accurately estimated (see Fig 8).

#### Contribution of genetic data to model assessment

It is well known that the predicted dynamics of spatio-temporal systems can be extremely sensitive to the choice of model, with consequent implications for the design of control strategies on the epidemic outbreaks [12, 26]. For example, studies of foot-and-mouth disease have cited the importance of selecting between a long-tailed spatial kernel against a localized spatial kernel [12, 32]. Other model-choice problems arise in relation to the parametric form of the distributions of incubation and infectious periods in models of measles [33, 34], and in relation to diseases such as smallpox [3, 35]. We show that increased availability of genetic data may increase the sensitivity over the mis-specification of the model, based on a latent-residual test recently developed [27]. For details see also S1 Text *:Contribution Genetic Data to Model Assessment* and Table S7 in S1 Text.

#### Testing the tolerance level of sub-sampling

In previous sections we have chosen the model parameters for simulated scenarios using values arising from practical considerations [12, 19, 20, 27]. In particular, the number of nucleotide bases and the mutation rates are chosen to lie within the respective ranges of these quantities for common animal viruses [19, 20, 36]. In this section we explore how the values/assumptions of some key model parameters may affect the level of sub-sampling for achieving a robust inference. As the duration and the rate of mutation are influential for the joint inference considered in this paper, we focus on exploring the effect of these two model quantities.

We consider pathogens with much smaller mutations rates (e.g. foot-and-mouth disease virus) than those we have considered in previous sections. Notably, results show that the estimations of the full set of model parameters and other dynamics are still feasible under the scenario with only 10% of sub-sampling (see S2 to S6 Figs), in contrast to 50% in previous sections. This could indicate that when mutation rates are higher, and transmitted sequences on exposures may be more diverse, higher rates of sampling the exposures may be required for robust inference. Also, we demonstrate that, in S7 and S8 Figs, a relatively small sub-sampling level (e.g. 20%) may be tolerated if the model parameters of the latent period distribution are assumed to be known.

#### Single-cluster epidemic and partial genome sequencing

We also consider the single-cluster scenario considered in many practical applications (e.g. [19, 20]). We compare the case of *partial genome sequencing* with the case where full genome sequencing is considered. Specifically we consider a (random) subset of the original set of 8000 sites of length *n* = 1000. The transmission graph and the model parameters can be accurately estimated and the effect of sub-sampling of exposures is similar to that observed in multiple-cluster scenarios (see S9 to S11 Figs). Comparison between S10 and S11 Figs demonstrates that a higher degree of sequencing of the genome gives rise to narrower credible intervals for *μ*_1_ and *μ*_2_ compared to the case with partial genome sequencing. It reveals that partial genome sequencing may be sufficient if the transmission graph and epidemiological model parameters are of primary interest as the quality of the estimation appears robust to reduction of the amount of sequencing of the genome.

To show that the increasing genetic data systematically provide extra information on the transmission dynamics, extensive simulation studies that consider alternative scenarios are conducted (see S1 Text *: Further Simulated Epidemics*, and Table S1 to S6 in S1 Text).

### Case Study: Spread of Foot-and-Mouth Disease Virus in UK (Darlington, Durham County, 2001)

In this section we apply our algorithm to a localized FMDV outbreak that occurred in the UK (Darlington, Durham County) in 2001, in which 12 infected premises (indexed here by the letters C-P), forming the so-called “Darlington cluster”, were observed and sampled to obtain one virus sequence for each premises with sequence length *n* = 8176 [9, 20]. The geographical locations, the sampling times and removal (i.e. culling) times of the infected premises were reported. Estimated onset dates of lesions were also provided by experts at the times of sampling. These data were previously analysed by [20] in one of the first important attempts, using a pseudo-likelihood approach, to jointly consider epidemiological and genetic data in an integrated framework. Note that, 3 additional premises were not included in previous analysis as these premises were believed not to be epidemiologically linked to the rest of the premises in the “Darlington cluster”. Here, for a more valid comparison, we analyse the same dataset using our methodology.

As in the section *Simulation Studies*, where we have tested our methodology with a much larger number of sites *N* = 150, we fit a spatial SEIR model to the data. In particular, we assume that sojourn times in classes E and I follow *Gamma*(*a*, *b*) characterized by the shape *a* and scale *b* and *Exp*(*μ*_*r*_) characterized by the mean *μ*_*r*_ respectively. The spatial kernel is assumed to be an exponentially-bounded kernel exp(−*κd*_*ij*_) (Refs [20]). The model is fitted to the data using the methods as described in *A Systematic Bayesian Integration Framework*. A single-cluster scenario has been assumed in [20]. To validate this assumption and demonstrate the generality of our framework, we allow multiple clusters in our analysis.

We consider whole genome sequencing in this section. The estimated onset dates of lesions provide important information on the starting dates of infectiousness for infected premises as these two dates were suggested to be close to each other [37]. To incorporate uncertainty in the estimated lesion onset dates, for each infected premises we allow the onset of infectiousness to vary within a 14-day interval centered at the estimated lesion onset date provided. It is noted that, given that the maximum of the estimated duration between lesion onset times and sampling times is 7 days, 14 days may represent a conservative upper bound of the estimation uncertainty.

#### Validating previous findings

**Fig 9:**
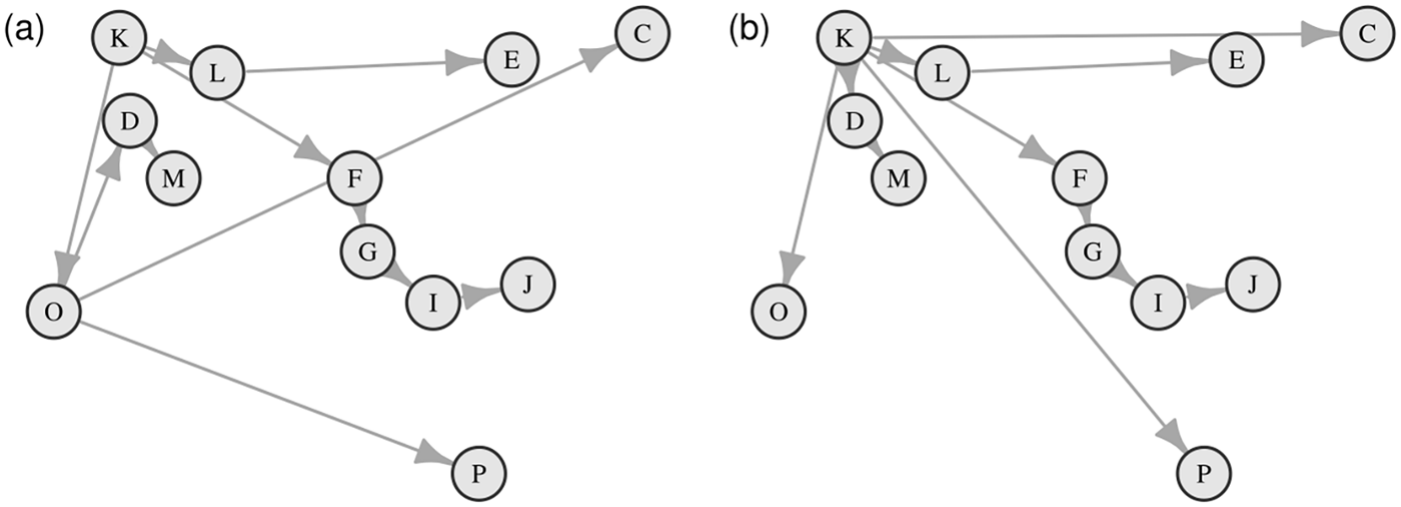
(a) The transmission graph with highest posterior probability, 0.89. (b) The transmission graph with the second highest posterior probability, 0.08. The same set of labels of premises used in [20] are adopted to facilitate comparison.

Fig 9 shows the transmission graphs with the highest and second highest posterior probability. We first notice that, although we fit a multiple-cluster model, our results validate the single-cluster assumption made by [20]. Similar to their analysis, premises *K* was also identified as the index case of the transmission with high posterior probability. The longest sequence of transmissions (i.e., *K* → *F* → *G* → *I* → *J*) coincides with their estimate. The most probable infection sources for premises *O* and *L* are the same in both analyses. The infecting sources of the remaining premises identified in [20] are not entirely consistent with our estimates. For example, the sources for premises *C* and *P* were only identical to ours in our second most probable transmission graph and the source for premises *M* was identified to be premises *O* instead of premises *D*. Nevertheless, the posterior distribution of the transmission graph which we obtain is broadly consistent with the earlier analysis, also reinforcing the argument made in [20] that the pseudo-likelihood approach may be sufficiently effective if the transmission network is of primary interest.

#### Improvements of inference

**Fig 10:**
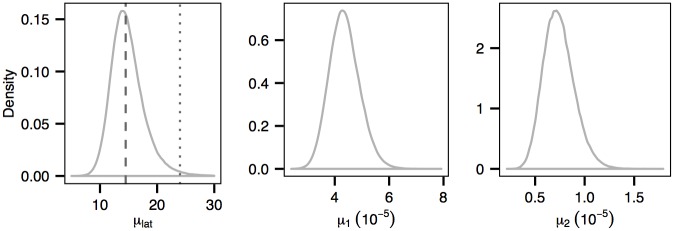
Posterior distributions of the mean latent period, denoted as *μ*_*lat*_, and of the transition rate *μ*_1_ and transversion rate *μ*_2_. The grey dashed line and the dotted line indicate the median value of *μ*_*lat*_ obtained from our analysis and from [20] respectively.

The mutation rates make a significant contribution to the likelihood and therefore to the joint inference of epidemic and evolutionary process. Application of our method enables us to estimate the mutation rates (Fig 10) which were assumed to be known in [20]. Note that we allow two types of mutation (transition and transversion) while previous analyses assumed a single aggregate mutation rate [9, 20]. Nevertheless, the orders of magnitude of our estimated mutation rates are consistent with the literature [9, 36]. It is noted that these estimated mutation rates are slightly lower than the smallest values assumed in the simulation study (see S12 Fig).

The typical value of the latent period (i.e., sojourn times in class E) of FMD suggested in the literature is around 5 days (with 95% confidence interval [1, 12]) [37–39]. However, with the same dataset, the median of the mean latent period was estimated in [20] to be much higher (24 days with 95% C.I. [17 days, 35 days]). These authors hypothesized that the over-estimation was likely due to the scenario that some of the infected premises in the data were actually infected by undetected infectious premises. Fig 10 shows the posterior distribution of the mean latent period obtained using our method. It suggests a significantly lower median value of the mean latent period, 14.2 days, compared with the previous estimate of 24 days. Although our estimated mean latent period is much closer to the range suggested in the literature it is nevertheless distinctly high, supporting the notion that undetected infected premises may play a role [20].

#### Sensitivity analysis: Inclusion of unreported susceptibles

**Fig 11:**
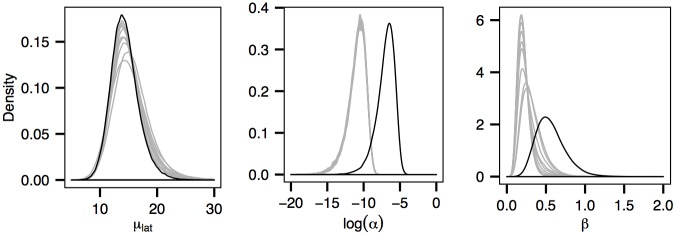
Posterior distributions of the mean latent period *μ*_*lat*_, the primary transmission rate *α* and the secondary transmission rate *β* obtained from fitting the model to 10 independently simulated datasets (grey curves) obtained by adding 300 randomly assigned susceptible premises. The posteriors corresponding to the case ignoring susceptibles are coloured in black. The values of primary transmission rate *α* are represented on the logarithmic scale for ease of comparison.

The number and locations of susceptible premises in the region were not reported and therefore were not considered in the earlier analysis [20]. In this section we investigate the effect of unreported susceptibles on estimation by randomly assigning 300 susceptible premises in a rectangular region (253 *km* ^2^) encompassing the sampled premises. The number of susceptible farms we choose ensures that the farm density in the area we consider is consistent with the crude farm density across Durham County [40, 41]. Note that the model dimension does not expand significantly after the inclusion as we are not required to consider genetic sequences on susceptible sites. Results show that most of the model parameters, except the primary and secondary transmission rates, are robust to the inclusion of significant numbers of susceptible sites (Fig 11). In particular, we notice that the mean latent period is only slightly affected. The posterior distribution of the transmission graph is largely unaffected (not shown here). Posterior distributions for the full set of model parameters are shown in S12 Fig.

## Discussion

In response to the increasing availability of genetic data from pathogens in epidemic outbreaks substantial progress has been made on the joint analysis of epidemiological and genetic data [9, 13–21]. However, existing approaches make use of approximations in modelling the epidemiological-evolutionary process, which in particular avoid inferring the *unobserved sequences transmitted* from donors to recipients upon infections or use approximate Bayesian inference to account for these sequences. These approximate approaches greatly reduce the computational challenges inherent in inferring the unobserved transmitted sequences, but only partially capture the joint epidemiological-evolutionary dynamics (Refs [23–26]) and may lead to less robust and accurate inference – for instance, the reconstruction of the transmission tree can be sensitive to priors chosen for some epidemiological parameters [16] and the latent period of a disease may be overestimated [20]. There is therefore a need to extend current approaches and develop a more systematic framework for the joint inference of these two coupled processes. Such a framework is useful to better understand the epidemic dynamic and to systematically characterise the importance of genetic data, which may yield useful insights for predicting, managing and controlling the epidemics [12, 25, 26].

We show that it is feasible to systematically integrate epidemiological and genetic data by devising an algorithm for jointly imputing the transmission graph and the transmitted sequences in a statistically sound Bayesian framework. Our key innovation is the development of an MCMC algorithm that allows for explicit representation and imputation of unobserved, transmitted sequences which in turns facilitates the use of realistic likelihood functions in the analysis. We have tested and validated this methodology via specifically-designed computer experiments (for details see S1 Text *:Validation of the Methodology*) and demonstrated its utility in a range of scenarios. We have tested our methods on epidemics with moderate size (*n*∼150) comparable to those used in practical applications [16, 18, 20], which should also suffice for example, providing insights into decision support during the early stage of a major outbreak. Also, the run-time is greatly reduced when we consider partial genome sequencing, but that this resulted in no material difference in the estimates of epidemiological parameters compared to using full genome sequencing (see S1 Text *:Computing Time and Other Benchmarks*).

Our results also have important implications for future study design. Using our methods, we characterise and quantify the effect of using a subset of genetic data from a number of important perspectives. First, generally speaking, both the epidemiological and evolutionary model parameters, including the transmission graph, are more accurately estimated when more genetic data are available. In particular, we show that the spatial transmission mechanism (i.e. the spatial kernel) can be estimated more precisely. The identification of the clusters of transmission helps the identification of risk factors and yields useful insights into devising effective control strategies [30, 31]. We show that, even if the transmission graph may not be well-identified at low levels of sub-sampling of sequences data, the clusters and the sites of primary infections can still be identified with good accuracy. We also show that the parameter values of mutation rates and latent period distributions can have some influence on the tolerance level of sub-sampling for achieving robust inference. Moreover, our results suggest that partial genome sequencing may be adequate if the epidemiological dynamic is of primary interest. Lastly, we demonstrate that genetic data can also facilitate model assessment using methods recently developed by the authors [27].

We show the practical usage of our framework by applying our methods to data on the FMD outbreak in 2001 in the UK, demonstrating both agreement with and improvement over previous findings. First, our results suggest a transmission graph broadly consistent with previous work [20], supporting the use of specific pseudo-systematic approaches [16, 20] when only the transmission graph is of primary interest. Also, our results validate the one-cluster assumption used in [20], which also demonstrates the flexibility of our (multiple-cluster) framework. On the other hand, we show that more realistic estimates of the latent period can be obtained, and mutation rates can also be estimated. This highlights the importance of explicitly taking into account the transmitted sequences for constructing a more accurate and integrated representation of the transmission dynamics, with the proximate goal of reliable prediction and the ultimate aim of effective management of disease outbreaks.

Our framework can readily accommodate more complicated models and be applied more generally, by relaxing a number of simplifying assumptions made in formulating the component models that we use in this paper. For instance, similar to many practical applications in the literature [16, 18, 20], we assume a dominant strain on an exposure at any time point. In doing so, we have not considered the within-host dynamic of the pathogens. By considering a single dominant strain, we assume that the transmitted strain in an infection event is a direct descendant of the strain transmitted in a previous transmission event involving the same donor. This assumption simplifies the structure of the tree that we need to consider (Ref [42]) and facilitates the design of the proposal distributions used for the joint updating of donor and transmitted strain which is fundamental to our algorithm. However, a within-host diversity model component can be included naturally, by at the same time specifying a distribution for selecting a transmitted strain among the multiple strains in a host. Similarly the assumption of having one master sequence *G*_*M*_ may be relaxed by treating *p* and *G*_*M*_ as nuisance parameters (see discussion in *Models and Methods*). For example, if suggested by empirical data or prior knowledge, one may allow for multiple distinct master sequences for different specified ranges/domains of time or space. We also note that the background/primary sequences are largely constrained by the sampled sequences, and the principal goal of including a primary infection model is to include more explicitly the primary sequences into our framework. Also, it is *not* required to assume a primary infection model when considering a single-cluster scenario.

Nevertheless, we have successfully demonstrated in this paper the feasibility of integrating systematically epidemiological and evolutionary processes using a methodology that allows explicit inference of both. Moreover, application to a real world problem demonstrates not only the practicality of this approach but also the added-value which it brings in terms of extracting information from available data.

## Supporting Information

S1 Text Supplementary information. We present the following supplementary information in S1 Text: 1) Validation of our methodology using computer experiments and a mathematical argument; 2) Supplementary details of the MCMC algorithm; 3) Supplementary details of assessing the contribution of genetic data to model assessment; 4) Further simulated epidemics; 5) Supplementary information on the evolutionary model and other supplementary information; 5) Supplementary tables Table S1–S7.
(PDF)

S1 Fig A computer experiment for validating the methodology. See also S1 Text. Comparisons between the posterior distributions of the coverage rates and of *κ* obtained from fitting two models, the full model (Scenario I) and the epidemic model (Scenario II), to the epidemic data (no sampled sequences).
(TIFF)

S2 Fig Inference for epidemics with lower mutation rates. Posterior distributions of the model parameters for the epidemic with lower mutation rates. Here we consider an epidemic with mutation rates that are in keeping with the FMD scenario. In particular, we set *β* = 8.0, *μ*_1_ = 10^−4^, *μ*_2_ = 5 × 10^−5^ with other model parameters being set to the values used for simulating the 3-cluster epidemic in the main text. In order to discern any resulting differences due to the change of mutation rates and genetic data, we consider a particular simulation yielding the same epidemic data as the 3-cluster epidemic. (a) Epidemiological parameters. (b) Evolutionary model parameters.
(TIFF)

S3 Fig Inference for epidemics with lower mutation rates. Posterior distributions of the overall coverage rate for the epidemic with lower mutation rates. Notice that, at the low sampling percentage (10%) the availability of genetic data may not increase significantly the coverage rates compared to the scenario without any samples.
(TIFF)

S4 Fig Inference for epidemics with lower mutation rates. Posterior individual coverage of the sources of infection for the epidemic with lower mutation rates in scenarios with sampling 100%, 50%, 10% and 0%. The symbol + indicates an actual primary case.
(TIFF)

S5 Fig Inference for epidemics with lower mutation rates. Posterior cluster identification rate of the infections (see definition in main text, within each actual cluster of the epidemic with lower mutation rates, in scenarios with sampling 100%, 50%, 10% and 0%.
(TIFF)

S6 Fig Inference for epidemics with lower mutation rates. Posterior (primary) ancestor identification rate of the infections (see definition in main text), within each actual cluster of the epidemic with lower mutation rates, in scenarios with sampling 100%, 50%, 10% and 0%.
(TIFF)

S7 Fig Inference for epidemics with a known latent period distribution. Posterior distributions of model parameters and the coverage rate from fitting the 3-cluster epidemic data with sampling proportion 20% (assuming the latent period distribution is known).
(TIFF)

S8 Fig Inference for epidemics with a known latent period distribution. Posterior distributions of model parameters and the cover rate from fitting the 6-cluster epidemic data with sampling proportion 20% (assuming the latent period distribution is known).
(TIFF)

S9 Fig Inference for single-cluster epidemics. Posterior distributions of the overall coverage rate (with the single-cluster epidemic). (a) *n* = 1000. (b) *n* = 8000. We assume *α* = 0.0004, *β* = 10.0 and other parameters are the same as those used for simulating the 3-cluster epidemic i the main text. We consider a particular simulation giving rise to a single-cluster epidemic.
(TIFF)

S10 Fig Inference for single-cluster epidemic. Violin plots showing the posterior distributions of the model parameters (with the single-cluster epidemic and number of bases *n* = 1000). Dashed lines represent the actual values of the model parameters. (a) Epidemiological parameters. (b) Evolutionary model parameters.
(TIFF)

S11 Fig Inference for single-cluster epidemic. Posterior distributions of the model parameters (with the single-cluster epidemic and number of bases *n* = 8000). Dashed lines represent the actual values of the model parameters. (a) Epidemiological parameters. (b) Evolutionary model parameters.
(TIFF)

S12 Fig Inclusion of susceptible farms for 2001 FMD outbreak in UK. Posterior distributions of the full set of model parameters obtained from fitting the model to 10 independently simulated datasets obtained by adding 300 randomly assigned susceptible premises to the 2001 FMD data (grey curves). The posteriors corresponding to the case when susceptibles are not considered are coloured in black. Non-informative flat priors are used for model parameters. Note that the posterior distributions of *p* appear to be almost the same as the prior (i.e., *U*(0, 1)). To facilitate comparison, the posteriors of *log*(*p* ^−1^) are presented and appear identical to an *Exp*(1)∼*log*(*U*(0, 1)^−1^) represented by the red dotted line, which suggests that the data are not sufficient for estimating *p* (see more discussion in S1 Text.
(TIFF)
