# Supplementary material for: A Systematic Bayesian Integration of Epidemiological and Genetic Data
Source: PLoS Comput Biol. 2015 Nov 23;11(11):e1004633. doi: 10.1371/journal.pcbi.1004633 (PMC4658172; doi:10.1371/journal.pcbi.1004633)
Supplement: S1 Text — We present the following supplementary information in S1 Text: 1) Validation of our methodology using computer experiments and a mathematical argument; 2) Supplementary details of the MCMC algorithm; 3) Supplementary details of assessing the contribution of genetic data to model assessment; 4) Further simulated epidemics; 5) Supplementary information on the evolutionary model and other supplementary information; 5) Supplementary tables Table S1–S7. (PDF) [file pcbi.1004633.s001.pdf]

S1 Text

Validation of the Methodology

Fitting a Full Model to Epidemic Data

In this section we aim to validate the implementations of the part of our algorithm concerned with the sampling of unobserved sequences and transmission graphs (fitting the six-cluster epidemic simulated in the main text). While estimation of the full set of model parameters is not feasible given insufficient genetic data (see main text), we consider a minimally sufficient case in which we compare the posterior distributions of the coverage rate and κ obtained respectively from two scenarios. In scenario I, we assume no genetic data and fit the full model (epidemic and genetic model). In scenario II, we assume no genetic data and fit only the epidemic model. Assuming other model parameters to be known, we also impute the times of exposures in both scenarios. In Scenario I we impute unobserved transmitted sequences, the master sequence and the transmission graphs which are the key components in our algorithm. Theoretically, the two scenarios should yield identical posterior distributions for the coverage rate and κ as the observed data are the same. S1 Fig. shows that the posterior distributions of the coverage rate and κ display no significant differences, which in turn supports the validity of our algorithm. Note that the minor difference between the posterior (cumulative) distributions of the coverage rate is likely to be caused by numerical rounding behaviours due to widely differing model dimensions and hence in the magnitudes of likelihood values. In fact, the posterior (non-cumulative) densities suggest very similar coverages rates $-$ in scenario I, the coverage rate has mean 0.68 and a standard deviation 0.027; in scenario II, the coverage rate has mean 0.68 and standard deviation 0.028.

Posterior Distribution of Parameter p for the FMD Outbreak (Darlington, 2001)

S12 Fig. shows that the posterior distributions of p are very similar to its prior. Heuristically speaking, under our prior assumptions, the data are only informative about p when there is evidence of multiple clusters (i.e., when there are multiple background sequences S_1,S_2,... derived from the master sequence G_M). Since single-cluster transmission graphs are strongly supported under the posterior distribution arising from our analysis (see main text *Case Study*), should our algorithm be efficient in exploring the sequence and tree space and correctly implemented, we should expect the posterior for p to be identical to its prior. This is straightforward to verify mathematically.

Proposition 0.1 *Conditioning on a single-cluster transmission graph and on each base of the master sequence G_M being drawn* a priori *uniformly from the set $\omega_N = \{A, C, G, U\}$, the posterior for p is identical to its prior.*

Proof Denoting $\pi(p)$ and $\pi(p|S)$ as the the prior and the posterior distribution (given a single sequence S initiates the epidemic) of p respectively, we have

$$\begin{aligned}\pi(p|S) &\propto \pi(p) \times \sum_{G_M} P(G_M) \times P(S|p, G_M)\\ &\propto \pi(p) \times \sum_{G_M} P(S|p, G_M) \quad (\because P(G_M) = constant)\\ &= \pi(p) \times \sum_{G_M} P(G_M|p, S)\\ &= \pi(p).\end{aligned}$$

The last equality holds as

$$\sum_{G_M} P(G_M|p, S) = 1. \quad \blacksquare$$

Note that if we condition on there being more than one cluster, the second equality does not hold (i.e., $P(S_1, S_2, \dots |p, G_M) \neq P(G_M|p, S_1, S_2, \dots)$).

Supplementary Details of the MCMC Algorithm

The model parameters and the unobserved quantities are updated sequentially (see below). In this section we give supplementary details of the algorithm not described in the main text.

Sampling of E_j

In the Part I of the description of the algorithm in the main text, the exposure time $E_j^{'}$ is proposed as a random draw

$$E_j^{'} \sim U(t_l, t_u). \tag{1}$$

If j is a primary infection we have

$$t_l = 0 \tag{2}$$

and

$$t_u = \begin{cases} \min\{t_j^s, I_j\}, & \text{if } j \text{ has an observed sequence sample (at } t_j^s\text{) and } j \in \chi_I, \\ t_j^s, & \text{if } j \text{ has an observed sequence sample and } j \notin \chi_I, \\ I_j, & \text{if } j \text{ has no observed sequence sample and } j \in \chi_I, \\ t_{max}, & \text{if } j \text{ has no observed sequence sample and } j \notin \chi_I. \end{cases} \tag{3}$$

In the case of $\psi_j \in \chi_I$, we have

$$t_l = I_{\psi_j} \tag{4}$$

and

$$t_u = \begin{cases} \min\{t_j^s, I_j, R_{\psi_j}\}, & \text{if } j \text{ has an observed sequence sample (at } t_j^s\text{) and } j \in \chi_I, \\ \min\{t_j^s, R_{\psi_j}\}, & \text{if } j \text{ has an observed sequence sample and } j \notin \chi_I, \\ \min\{I_j, R_{\psi_j}\}, & \text{if } j \text{ has no observed sequence sample and } j \in \chi_I, \\ R_{\psi_j}, & \text{if } j \text{ has no observed sequence sample and } j \notin \chi_I. \end{cases} \tag{5}$$

When $\psi_j \notin \chi_R$, Equation 5 reduces to Equation 3. In the Part II of the description of the algorithm in the main text, $E_j^{'}$ is proposed in the same manner with ψ_j now being replaced by $\psi_j^{'}$. The reader is reminded that the sampling of $E_j^{'}$ is only a part of the joint sampling procedures described in the main text.

Sampling of I_j

To incorporate some uncertainty in the onset of infectiousness, I_j is assumed known within a range. Let t_o denote the actual time of symptom onset. Then we assume that I_j is known only within a range $t_o \pm D$. For simulation studies, we assume t_o to be the true I_j and $D = 0.6$. Taking account of the additional constraint that $E_j < I_j < R_j$, we propose $I_j^{'}$ uniformly between t_a and t_b where

$$t_a = \max\{E_j, t_o - D\} \tag{6}$$

and

$$t_b = \min\{R_j, t_o + D\}. \tag{7}$$

R_j is replaced by t_{max} if $j \notin \chi_R$.

Sampling of $G_{1,j}$

In addition to the joint sampling of $E_j^{'}$ and $G_{1,j}^{'}$, separate updating of $G_{1,j}^{'}$ is necessary to explore thoroughly the domain of $\boldsymbol{G}$. We implement a simple updating algorithm for proposing $G_{1,j}^{'}$ – for an individual $j \in \chi_E$, each nucleotide base $G_{1,j}^{'i}$ is sampled uniformly from the set $\omega_N = \{A, C, G, T\}$.

Sampling of $\boldsymbol{\theta}$

Each parameter in $\boldsymbol{\theta} = (\alpha, \beta, a, b, \gamma, \eta, \kappa, p, \mu_1, \mu_2)$ is updated sequentially with a standard random-walk Metropolis-Hastings algorithm. For example, a new parameter value α' is proposed from a normal distribution centered on the current value of α

$$\alpha' \sim N(0, \rho^2) \tag{8}$$

where ρ controls the step-size of the random-walk.

Sampling of Cryptic Exposures

Denote ω_C as the set of exposed individuals who do not have an observed sample and are not in χ_I. We refer to $j \in \omega_C$ as to a *cryptic* exposure. We incorporate j in our framework by imputing the sequence transmitted to j. Allowing cryptic exposures requires a 'swap' of individuals between the sets ω_C and χ_S and a transmitted sequence needs to be imputed when an individual from χ_S moves to ω_C. After the individual to be swapped has been proposed, the sequence is imputed in a similar manner to Part II of the algorithm described in the main text. The acceptance probability is similar to that in Part II of the algorithm described in the main text with an additional term that accounts for the 'swapping' probability [1].

Initialisation of the Transmission Graph $\boldsymbol{\psi}$

When only a subset of individuals $j \in \chi_E$ have an observed sequence sample, the choice of the starting value of $\boldsymbol{\psi}$ becomes important for the rate of convergence of the Markov chain. In this case, we sample the starting value $\boldsymbol{\psi}_0$ from the marginal posterior distribution of $\boldsymbol{\psi}$, $P_e(\boldsymbol{\psi})$, obtained from only fitting the epidemic model to the epidemic data using standard data-augmentation methods. Effectively, we set $g(\cdot) = h(\cdot) = 1$ in the likelihood function (main text) and do not attempt to impute the unobserved sequences.

Sampling and Initialisation of G_M

The master sequence G_M determines the source sequence for a particular cluster and the choice of its initial value in the MCMC algorithm is very important. Specifically, we choose the first observed sample in the population as the initial value. We implement a simple updating algorithm similar to the updating of $G_{1,j}$ – each nucleotide base in $G_M'^i$ is proposed uniformly from the set $\omega_N \setminus G_M^i$.

Initial Values of Other Parameters

In the application to FMDV, we initially set $\alpha = 0.0002$, $\beta = 3.0$, $a = 2.0$, $b = 2.0$, $\mu = 8.0$, $\kappa = 0.1$, $\mu_1 = 1e - 04$ and $\mu_2 = 5e - 05$ as the initial values. In the simulation studies, each parameter in $\boldsymbol{\theta}$ is initialised to be one half of its true value. An initial value of I_j, $j \in \chi_I$, is randomly drawn within a range $t_o \pm D$. Set the initial χ_E be χ_I (i.e., no cryptic exposures). Let $\omega_\psi = \{j \in \chi_I | I_j \leq t_u\}$ be the set of potential sources for a particular individual $i \in {\chi_E}^{-1}$. The source of i, ψ_i, is then chosen uniformly from ω_ψ. Note that in the case of fitting the full model, a candidate drawn from $P_e(\boldsymbol{\psi})$ is set to be ψ_i if it is also in χ_ψ (see also main text). After initialising the transmission network and times of events (i.e., E_j and I_j), the transmitted sequences are initialised sequentially in the order of E_j according to the evolutionary model specified by Equation 2 in the main text.

Computing Time and Other Benchmarks

The MCMC algorithm was coded in the C++ language (executed on a system with an Intel(R), i7-2600, 3.40GHz CPU). To provide a benchmark, we report the computing time and some key features of the Markov chain from the simulated single-cluster example where full genome sequencing and full sampling of exposures were considered (i.e., population size $N = 150$, sequence length $n = 8000$ and sampling proportion =100%).

Convergence and mixing of the chain were assessed on the basis of visual inspection of trace plots. MCMC output is a chain of autocorrelated samples, and a common measure of mixing and the size of independent samples is the so-called *effective sample size (Eff)* which aims at "un-coupling" the effect of autocorrelation (it is often advised not to stop the MCMC with $Eff < 100$). The effective sample size of a parameter θ is commonly defined as $Eff(\theta) = S/(1 + 2\sum_k \rho_k(\theta))$, where S is the number of posterior samples and $\rho_k(\theta)$ is the autocorrelation at lag k (the sum is usually truncated at lag k when $\rho_k(\theta) < 0.05$). The effective sample sizes for individual model parameters were computed using a package [2] available in the statistical software R. We obtained a converged and well-mixed chain with a reasonable effective sample size (obtained from 400,000 iterations after 50,000 burn-in). The computing time was 63803.28 seconds (17.7 hours) which is considered to be practical and efficient [3, 4]. The effective size was $Eff_{\boldsymbol{\theta}} = (286, 912, 998, 5380, 1950, 7416, 9945, 30133)$ with elements corresponding to parameters in $\boldsymbol{\theta} = (\beta, a, b, \gamma, \eta, \kappa, \mu_1, \mu_2)$. We also report that the computing time is greatly reduced (i.e., 2.3 hours) in the case of partial genome sequencing with $n = 1000$ − this has practical implications, for the estimates of most epidemiological parameters obtained from using partial genome sequencing have no material difference compared to using full genome sequencing (S10 Fig. −S11 Fig.). We also note that our code may be parallelised fairly easily for a potentially significant reduction in run time, using multi-core computers that are becoming more common nowadays; for example, mutations nucleotide sites are assumed to be independent of each other, which can be utilised for parallelisation in a straightforward manner using popular platforms like MPI or OpenMP.

Acceptance Probabilities

The acceptance probability of a proposed parameter value $\theta_i^{'}$ with current value θ_i is

$$p_a = \min\{1, \frac{L(\boldsymbol{\theta}^{'}; \boldsymbol{z})}{L(\boldsymbol{\theta}; \boldsymbol{z})} \times \frac{P(\theta_i^{'})}{P(\theta_i)} \times \frac{q(\theta_i|\theta_i^{'})}{q(\theta_i^{'}|\theta_i)}\} \tag{9}$$

where $P(\theta_i)$ is the *prior distribution* of θ_i and $q(\theta_i^{'}|\theta_i)$ *proposal distribution* of $\theta_i^{'}$ given the current value θ_i. The probability of accepting a proposal to a component of the augmented data $\boldsymbol{z}_i^{\prime}$ is similar. In most of the cases, q is a symmetric proposal distribution and hence the *proposal ratio* (e.g., $\frac{q(\theta_i|\theta_i^{'})}{q(\theta_i^{'}|\theta_i)}$) reduces to 1, simplifying the problem. However, when the proposal density is less straightforward the proposal ratio must be treated explicitly.

We describe in detail the computation of the proposal ratio for the joint sampling of $E_j^{'}$ and $G_{1,j}^{'}$ described in Part I of the algorithm in the main text. As an illustration, we consider only the case where G_p and G_f are both defined. We have to compute the *forward proposal probability* (i.e., the denominator)

$$q(E_j^{'}, G_{1,j}^{'}|E_j, G_{1,j}) = q_1(E_j^{'}|\boldsymbol{E}) \times q_2(G_{1,j}^{'}|E_j^{'}, \boldsymbol{E}, \boldsymbol{G}) \tag{10}$$

and the *backward proposal probability* (i.e., the numerator)

$$q(E_j, G_{1,j}|E_j^{'}, G_{1,j}^{'}) = q_1(E_j|\boldsymbol{E}') \times q_2(G_{1,j}|E_j, \boldsymbol{E}', \boldsymbol{G}'). \tag{11}$$

As ψ_j is unchanged the domains of E_j and $E_j^{'}$ are identical and we have

$$\frac{q_1(E_j|\boldsymbol{E}')}{q_1(E_j^{'}|\boldsymbol{E})} = 1. \tag{12}$$

We also have

$$q_2(G_{1,j}^{'}|E_j^{'}, \boldsymbol{E}, \boldsymbol{G}) = p_f^{m_f} \times (1 - p_f)^{n - m_f} \tag{13}$$

where m_f is the number of nucleotides on G_f which match the nucleotide in the corresponding position of $G_{1,j}^{'}$. The quantity $q_2(G_{1,j}|E_j, \boldsymbol{E}', \boldsymbol{G}')$ is similarly computed by considering the reverse move. In particular, we must re-define G_p and G_f as the direction of change of time is reversed. The proposal ratio for the joint sampling procedure in Part II of the algorithm in the main text can be easily

computed in a similar fashion. In this case we must take account of the difference in the domains of E_j and $E_j^{'}$ in the respective proposal distributions, and the ratio of probabilities of proposing ψ_j and ψ_j' as described in Part II of the algorithm in the main text. Non-informative uniform priors with "unrealistically" wide intervals are specified for all model parameters. For example, the prior for mutation rates is $U(0, 0.1)$, the mean latent period has a prior $U(0, 50)$ and the secondary transmission rate β has a prior $U(0, 50)$.

Contribution Genetic Data to Model Assessment

The authors have shown that effective model assessment of a general spatio-temporal model may be achieved by proposing suitably designed non-centred parameterization schemes and imputing the corresponding residuals, whose sampling distributions are known, in such a manner that posterior distributions are sensitive to mis-specifications of particular components of the model [6]. Here we investigate how the genetic data may help in assessing, in particular, the goodness-of-fit of a specified *spatial kernel* by utilizing the so-called *Infection-link Residual (ILR)*.

The set of ILR, hereinafter denoted as $\boldsymbol{r} = \{r_1, r_2, \cdots, r_{n_e}\}$ where n_e is the total number of exposures, uniquely determines the respective *infection link* (i.e., source of infection) for every exposure. The distribution of $\boldsymbol{r}$ can be shown to be $U(0, 1)$ under their construction scheme and the model assumption given by Equation (1) in the main text and is independent *a priori* of the form of the spatial kernel. Its posterior samples, hereinafter denoted as $\tilde{\boldsymbol{r}}$, can be easily imputed in standard data augmentation algorithms such as Markov chain Monte Carlo (MCMC) by inverting the construction procedures of ILR and imputing the infection links. On applying a classical test to $\tilde{\boldsymbol{r}}$ for its compliance with $U(0, 1)$, a posterior distribution of *p-values* is generated from which the evidence against the model assumption can be discerned. Specifically, we measure the evidence against the model by $\pi(P(\tilde{\boldsymbol{r}}) < 0.05|\boldsymbol{y})$, the proportion of the posterior p-values which are less than 0.05. The *Anderson-Darling* hypothesis test [5] is adopted (for details see [6]). We consider the six-cluster epidemic data mentioned in the main text.

We consider fitting three forms of spatial kernel:

- An exponentially-bounded kernel (Kernel A): $K(d_{ij}, \kappa_1) = exp(-\kappa_1 d_{ij})$;
- A power-law kernel (Kernel B): $K(d_{ij}, \kappa_2) = d_{ij}^{-\kappa_2}$;
- A Cauchy-type kernel (Kernel C): $K(d_{ij}, \kappa_3) = \frac{1}{\kappa_3\{1+(\frac{d_{ij}}{\kappa_3})^3\}}$.

It is noted that Kernel A is the actual spatial kernel used in the simulations.

In the main text we have shown that increased availability of genetic data improves the estimation of the transmission graph. Given that the imputations of ILR rely on the imputed infection links (equivalently the transmission graph), increased availability of genetic data may potentially increase the sensitivity of the test based on imputed ILR over the mis-specification of the model. **Table S7** shows that this improvement of sensitivity is indeed achieved.

Further Simulated Epidemics

In this section we consider 15 random independent replicates of epidemics. Specifically we simulate 5 epidemics using each of the 3 sets of the model parameters where multiple-cluster scenarios were investigated. All the epidemics considered here are of more than one cluster. To recap, compared to the *first set* of model parameters, the *second set* of model parameters is characterized by a higher background transmission rate and hence is expected to give rise to epidemics exhibiting higher numbers of clusters than those generated from the first and third set of model parameters. The *third set* of model parameters is characterized by the lower mutation rates which match the foot-and-mouth disease scenario.

For epidemics simulated from the first and second sets of model parameters, we compare the estimation performance at sampling levels 100%, 50% and 0% of exposures. For epidemics simulated

from the third set of model parameters, we compare the estimation performance at sampling levels 100%, 10% and 0% of exposures.

In the main text it is observed that posterior uncertainty in the model parameter estimates tends to increase as the sampling % drops with this effect appearing most dominant for the secondary transmission rate β and the spatial kernel parameter κ. Tables S1−S3, which show the sample means and standard deviations of the posterior samples of β and κ, suggest similar findings. Note that for the third set (characterized by significantly lower mutation rates and showing a higher tolerance to level of sub-sampling) we have considered sampling level 10% (instead of 50%) obtaining results similar to the 0% setting.

Tables S4−S6 show the absolute difference between the number of clusters obtained from the posterior samples and the actual number of clusters, denoted as ΔN_c. They also show the number of different bases (out of 1,000) between the imputed master sequence G_M and the actual ones, denoted as ΔM, and the overall coverage rate obtained from the posterior samples. Similar to the findings shown in the main text, it is observed that ΔN_c and ΔM in general increase when the sampling percentage reduces. In the case where no genetic data are available the mean of ΔN_c, and its degree of variation, are quite considerable. Also, comparison of the values of ΔM from Table S4 and Table S5 reveals that the estimation of G_M may become more reliable as the number of clusters increases. It is observed that when there are fewer than 3 clusters in the actual epidemic (e.g., Replicate 1, Replicate 3 and Replicate 5 in Table S4), ΔM becomes considerably larger. The overall coverage rate increases with the sampling percentage and becomes less dispersed.

A Markov Process to Model the Evolutionary Process

A continuous-time Markov process with states (i.e., nucleotide bases) taking values in the set $\omega_N = \{A, C, G, U\}$ can be defined to model the nucleotide substitution process. Let μ_{xy} be the transition rate between state $x \in \omega_N$ and $y \in \omega_N$. Moreover, let $\mathbf{P}(\triangle t)$ be the *transition probabilities matrix* whose entry $p_{xy}(\triangle t)$ is the probability of transition to state y after arbitrary evolutionary time $\triangle t$, given the initial state x.

The particular form of $\mathbf{P}(\triangle t)$ depends on the model assumptions. For details of the derivation of $\mathbf{P}(\triangle t)$ see [7]. For example, the simplest form of a nucleotide substitution model (the *Jukes-Cantor model*) assumes that the transition rates between any pair of nucleotide bases are the same (i.e., $\mu_{xy} = \mu$, $x \neq y$). Under the JC model,

$$\mathbf{P}(\triangle t) = \begin{pmatrix} 1-3a_t & a_t & a_t & a_t \\ a_t & 1-3a_t & a_t & a_t \\ a_t & a_t & 1-3a_t & a_t \\ a_t & a_t & a_t & 1-3a_t \end{pmatrix}$$

where

$$a_t = \frac{1 - e^{-4\mu\triangle t}}{4}. \tag{14}$$

The *Kimura model* we adopt [7] allows different rates for transition and transversion. Let μ_1 and μ_2 be the rates of transition and transversion respectively. Then under the Kimura model

$$p_{xx}(\triangle t) = 0.25 + 0.25e^{-4\mu_2\triangle t} + 0.5e^{-2(\mu_1+\mu_2)\triangle t}, x \in \omega_N \tag{15a}$$

$$p_{xy}(\triangle t) = \begin{cases} 0.25 + 0.25e^{-4\mu_2\triangle t} - 0.5e^{-2(\mu_1+\mu_2)\triangle t}, & \text{for } x \neq y \text{ and it is a transition.} \\ 0.25 - 0.25e^{-4\mu_2\triangle t}, & \text{for } x \neq y \text{ and it is a transversion.} \end{cases} \tag{15b}$$

Note that the notation used to denote transition probabilities differs from that used in Equation (2) in the main text. The notation $p_{xy}(\cdot)$ used here is more intuitive in the context of a matrix.

Signum Function

The signum function of a real number t is defined as follows:

$$sgn(t) = \begin{cases} -1, & \text{if } t < 0, \\ 0, & \text{if } t = 0, \\ +1, & \text{if } t > 0. \end{cases} \tag{16}$$

Table S1: **Independent replicates of multiple-cluster epidemics simulated from the 1st *set* of model parameters with $\alpha = 0.0004$, $\beta = 8$, $\kappa = 0.02$, $a = 10$, $b = 0.5$, $\gamma = 2.0$, $\eta = 2.0$, $p = 0.01$, $\mu_1 = 0.002$ and $\mu_2 = 0.0005$.** Sample means and standard deviations of the posterior samples of β and κ obtained from fitting the model to 5 independent replicates of multiple-cluster epidemics simulated from the *first set* of model parameters.

	$\beta = 8.0$			$\kappa = 0.02$		
Sampling%	100%	50%	0%	100%	50%	0%
Replicate 1	8.39 (1.13)	7.38 (1.12)	10.08 (4.29)	0.018 (0.001)	0.018 (0.0012)	0.021 (0.0029)
Replicate 2	6.91 (0.82)	7.45 (1.02)	10.91 (8.73)	0.019 (0.0011)	0.019 (0.0012)	0.021 (0.0045)
Replicate 3	7.92 (1.01)	8.92 (1.34)	12.64 (6.71)	0.019 (0.0011)	0.019 (0.0012)	0.022 (0.0035)
Replicate 4	7.64 (1.02)	8.28 (1.29)	12.73 (7.95)	0.02 (0.0012)	0.021 (0.0014)	0.024 (0.0048)
Replicate 5	8.90 (1.27)	10.02 (1.96)	9.57 (4.78)	0.02 (0.0012)	0.021 (0.0015)	0.022 (0.0031)

Table S2: **Independent replicates of multiple-cluster epidemics simulated from the 2nd *set* of model parameters, which is characterized by a higher background transmission rate and higher mutation rates compared to the first set of model parameters.** Sample means and standard deviations of the posterior samples of β and κ obtained from fitting the model to 5 independent replicates of multiple-cluster epidemics simulated from the *second set* of model parameters with $\alpha = 0.002$, $\beta = 8.0$, $\kappa = 0.02$, $a = 10$, $b = 0.5$, $\gamma = 2.0$, $\eta = 2.0$, $p = 0.01$, $\mu_1 = 0.003$ and $\mu_2 = 0.001$.

	$\beta = 8.0$			$\kappa = 0.02$		
Sampling%	100%	50%	0%	100%	50%	0%
Replicate 1	8.29 (1.17)	8.21 (1.57)	6.21 (2.17)	0.021 (0.0012)	0.020 (0.0015)	0.018 (0.0023)
Replicate 2	8.13 (1.11)	7.89 (1.25)	8.47 (4.11)	0.021 (0.0013)	0.019 (0.0013)	0.020 (0.0033)
Replicate 3	8.16 (1.09)	10.58 (1.98)	14.91 (7.87)	0.019 (0.0012)	0.020 (0.0013)	0.023 (0.0036)
Replicate 4	8.60 (1.25)	8.88 (1.62)	8.75 (4.91)	0.021 (0.0012)	0.021 (0.0015)	0.020 (0.004)
Replicate 5	7.80 (1.06)	9.12 (1.45)	10.22 (5.83)	0.021 (0.0012)	0.021 (0.0013)	0.022 (0.0036)

Table S3: **Independent replicates of multiple-cluster epidemics simulated from the 3rd *set* of model parameters, characterized by lower mutation rates, compared to the first set of model parameters.** Sample means and standard deviations of the posterior samples of β and κ obtained from fitting the model to 5 independent replicates of multiple-cluster epidemics simulated from the *third set* of model parameters with $\alpha = 0.0004$, $\beta = 8.0$, $\kappa = 0.02$, $a = 10$, $b = 0.5$, $\gamma = 2.0$, $\eta = 2.0$, $p = 0.01$, $\mu_1 = 10^{-4}$ and $\mu_2 = 5 \times 10^{-5}$.

	$\beta = 8.0$			$\kappa = 0.02$		
Sampling%	100%	10%	0%	100%	10%	0%
Replicate 1	9.79 (1.61)	7.36 (1.52)	10.04 (4.33)	0.019 (0.0013)	0.018 (0.0016)	0.021 (0.003)
Replicate 2	9.69 (1.55)	9.76 (2.59)	13.99 (8.9)	0.021 (0.0013)	0.022 (0.002)	0.024 (0.0043)
Replicate 3	8.59 (1.25)	8.22 (1.71)	12.60 (6.62)	0.019 (0.0012)	0.019 (0.0017)	0.022 (0.0035)
Replicate 4	9.02 (1.41)	8.92 (2.47)	12.73 (7.95)	0.022 (0.0014)	0.023 (0.0023)	0.024 (0.005)
Replicate 5	9.02 (1.36)	8.82 (1.95)	13.46 (7.99)	0.021 (0.0014)	0.021 (0.0018)	0.023 (0.0036)

Table S4: **Independent replicates of multiple-cluster epidemics simulated from the 1st *set* of model parameters.** Define ΔM to be the number of bases differing between the imputed master sequence G_M and the true sequence used in the simulation, and similarly ΔN_c to be the difference of number of clusters between actual and inferred quantities. This tables shows the posterior summaries of ΔN_c, ΔM and the coverage rate, obtained from fitting the model to 5 independent replicates of multiple-cluster epidemics simulated from the *first set* of model parameters. The mean values are followed by the standard deviations in brackets.

	ΔN_c			ΔM		$Coverage$(%)		
Sampling%	100%	50%	0%	100%	50%	100%	50%	0%
Replicate 1	0.01 (0.1)	0.18 (0.44)	1.60 (3.01)	19.3 (3.32)	19.0 (3.10)	99.9 (0.28)	83 (1.9)	58.5 (3.3)
Replicate 2	0.0 (0.05)	0.35 (0.61)	2.60 (4.59)	0.26 (0.51)	1.0 (0.84)	98.8 (0.31)	87.7 (1.8)	65.7 (4.6)
Replicate 3	0.04 (0.19)	0.22 (0.47)	0.95 (3.04)	24.7 (2.53)	29.37 (2.6)	99.7 (0.39)	85 (2.2)	62.7 (4.3)
Replicate 4	0.07 (0.25)	0.41 (0.62)	1.59 (3.27)	0.03 (0.17)	0.09 (0.28)	98.6 (0.50)	83.3 (2.2)	62.8 (4.1)
Replicate 5	0.0 (0.07)	0.25 (0.52)	1.03 (2.58)	14.3 (2.8)	13.1 (2.6)	99.9 (0.17)	82.8 (2.5)	61.4 (4.1)

Table S5: **Independent replicates of multiple-cluster epidemics simulated from the 2nd *set* of model parameters.** Posterior summaries of ΔN_c, ΔM and the coverage rate obtained from the posterior samples from fitting the model to 5 independent replicates of multiple-cluster epidemics simulated from the *second set* of model parameters, which is characterized by a higher background transmission rate and higher mutation rates compared to the first set of model parameters. The mean values are followed by the standard deviations in brackets. Note that, the master sequence G_M is estimated very accurately due to the larger number of clusters in this set of model parameters.

	ΔN_c			ΔM		$Coverage$(%)		
Sampling%	100%	50%	0%	100%	50%	100%	50%	0%
Replicate 1	0.12 (0.34)	2.11 (1.29)	3.44 (4.14)	0.0 (0.0)	0.0 (0.0)	98.3 (0.57)	83.7 (2.2)	63.3 (4.5)
Replicate 2	0.01 (0.1)	0.50 (0.73)	2.87 (4.58)	0.0 (0.0)	0.0 (0.0)	99.7 (0.36)	85.2 (2.1)	60.7 (4.3)
Replicate 3	0.14 (0.35)	1.18 (0.95)	2.43 (5.67)	0.0 (0.0)	0.0 (0.0)	99.1 (0.39)	75.5 (2.2)	61.6 (4.4)
Replicate 4	0.0 (0.0)	1.17 (1.09)	3.89 (4.94)	0.0 (0.0)	0.0 (0.0)	99.6 (0.4)	81.8 (2.3)	57.7 (4.7)
Replicate 5	0.0 (0.0)	2.20 (1.51)	2.77 (4.07)	0.0 (0.0)	0.0 (0.0)	99.2 (0.35)	79.8 (2.2)	65.2 (3.9)

Table S6: **Independent replicates of multiple-cluster epidemics simulated from the 3rd *set* of model parameters.** Posterior summaries of ΔN_c, ΔM and the coverage rate obtained from the posterior samples from fitting the model to 5 independent replicates of multiple-cluster epidemics simulated from the *third set* of model parameters characterized by lower mutation rates, compared to the first set of model parameters, to reflect beliefs regarding FMD virus. The mean values are followed by the standard deviations in brackets.

	ΔN_c			ΔM		$Coverage$(%)		
Sampling%	100%	10%	0%	100%	10%	100%	10%	0%
Replicate 1	0.01 (0.115)	0.61 (0.82)	1.54 (3.18)	12.06 (2.4)	12.0 (2.3)	89.4 (2.0)	60.3 (3.3)	58.5 (3.4)
Replicate 2	0.04 (0.20)	1.2 (1.15)	2.17 (3.58)	0.48 (0.50)	1.56 (0.66)	92.2 (1.7)	68 (3.0)	65.7 (3.7)
Replicate 3	0.02 (0.15)	0.49 (0.83)	0.88 (2.79)	16.93 (2.65)	18.38 (3.13)	93.6 (1.6)	66.4 (3.3)	62.6 (3.9)
Replicate 4	0.12 (0.35)	0.75 (0.56)	2.09 (2.59)	0.0 (0.0)	0.71 (0.71)	93.2 (1.6)	67.1 (3.3)	62.7 (4.0)
Replicate 5	0.0 (0.0)	0.32 (0.57)	0.74 (1.90)	4.89 (1.37)	5.71 (1.49)	94.9 (1.7)	68.1 (3.7)	64.5 (4.0)

Table S7: **Contribution of genetic data to model assessment.** Values of $\pi(P(\widetilde{\boldsymbol{r}}) < 0.05|\boldsymbol{y})$, where $\boldsymbol{y}$ is the observed data, estimated from 2,000 posterior samples of ILR computed from the 6-cluster epidemic under different model assumptions regarding the spatial kernel, with Kernel A being the one used in the simulation.

	$\pi(P(\widetilde{\boldsymbol{r}}) < 0.05\|\boldsymbol{y})$			
Sampling	100%	80%	50%	0%
Kernel A	10%	10%	9%	13%
Kernel B	50%	39%	36%	28%
Kernel C	100%	100%	100%	78%

References

[1] Gibson GJ, Renshaw E. Estimating parameters in stochastic compartmental models using Markov chain methods. Mathematical Medicine and Biology. 1998;15(1):19–40.

[2] Plummer M, Best N, Cowles K, Vines K. CODA: Convergence Diagnosis and Output Analysis for MCMC. R News. 2006;6(1):7–11. Available from: http://CRAN.R-project.org/doc/Rnews/.

[3] Ster IC, Singh BK, Ferguson NM. Epidemiological inference for partially observed epidemics: the example of the 2001 foot and mouth epidemic in Great Britain. Epidemics. 2009;1(1):21–34.

[4] Morelli MJ, Thébaud G, Chadœuf J, King DP, Haydon DT, Soubeyrand S. A Bayesian inference framework to reconstruct transmission trees using epidemiological and genetic data. PLoS Computational Biology. 2012;8:e1002768.

[5] Lewis PA. Distribution of the Anderson-Darling statistic. The Annals of Mathematical Statistics. 1961;p. 1118–1124.

[6] Lau MSY, Marion G, Streftaris G, Gibson GJ. New model diagnostics for spatio-temporal systems in epidemiology and ecology. J R Soc Interface. 2014;11:20131093.

[7] Yang Z. Computational molecular evolution. vol. 284. Oxford: Oxford University Press; 2006.
