# Supplementary figures and images for: A Systematic Bayesian Integration of Epidemiological and Genetic Data
Source: PLoS Comput Biol. 2015 Nov 23;11(11):e1004633. doi: 10.1371/journal.pcbi.1004633 (PMC4658172; doi:10.1371/journal.pcbi.1004633)

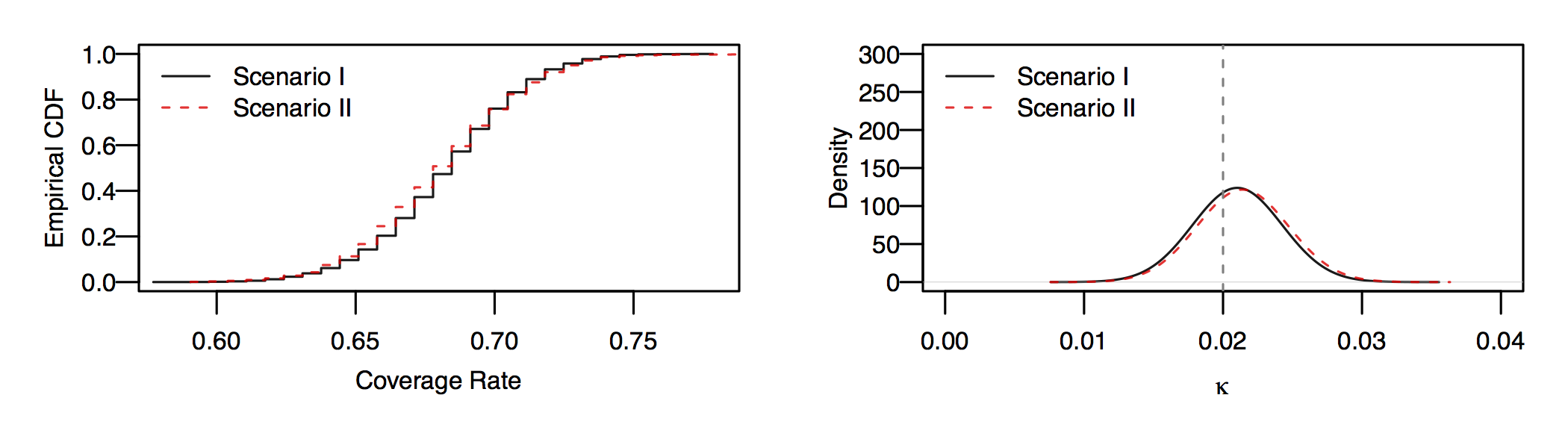

Supplement: S1 Fig — Comparisons between the posterior distributions of the coverage rates and of κ obtained from fitting two models, the full model (Scenario I) and the epidemic model (Scenario II), to the epidemic data (no sampled sequences). (TIFF) [file pcbi.1004633.s002.tiff]

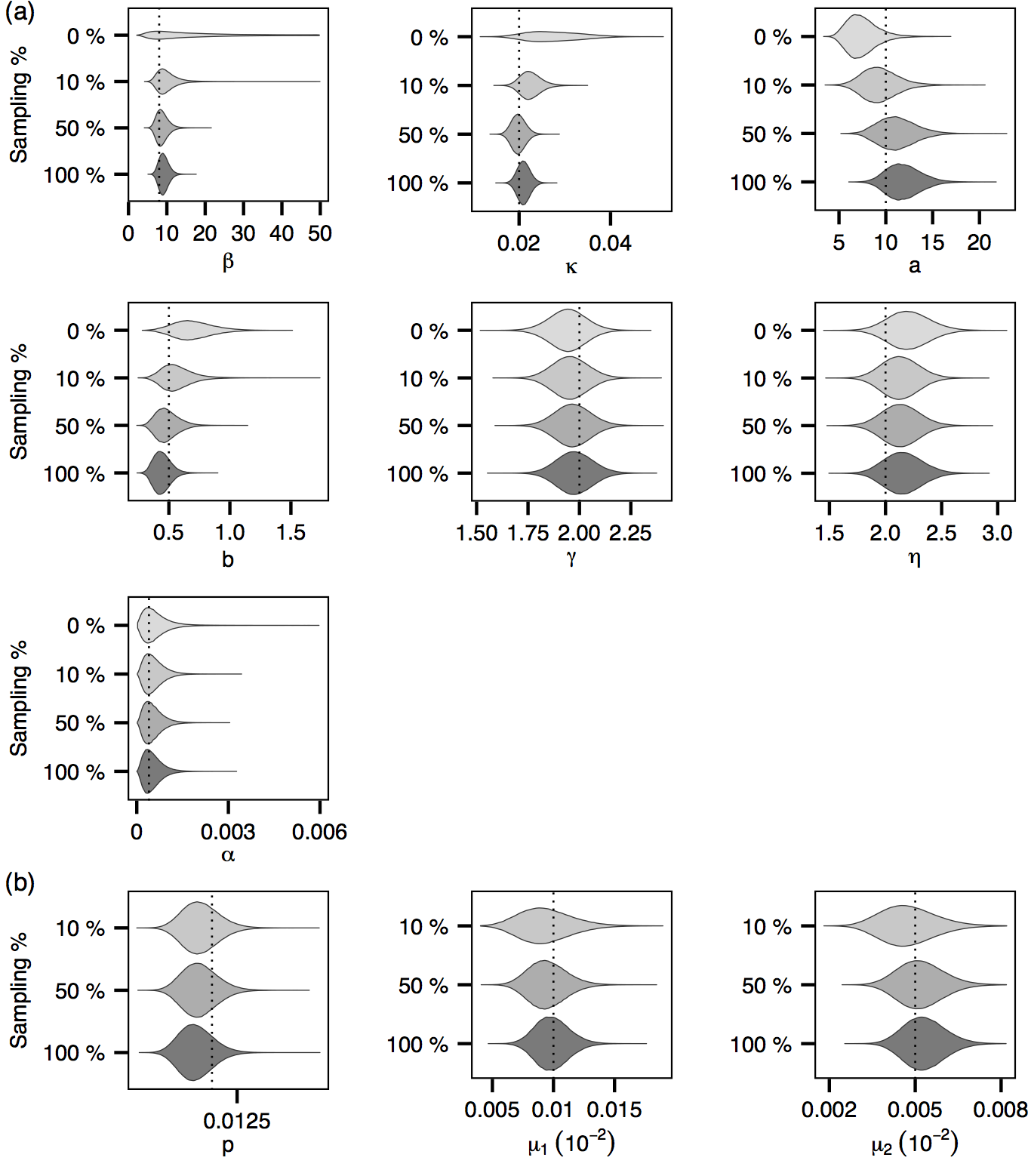

Supplement: S2 Fig — Posterior distributions of the model parameters for the epidemic with lower mutation rates. Here we consider an epidemic with mutation rates that are in keeping with the FMD scenario. In particular, we set β = 8.0, μ 1 = 10−4, μ 2 = 5 × 10^−5^ with other model parameters being set to the values used for simulating the 3-cluster epidemic in the main text. In order to discern any resulting differences due to the change of mutation rates and genetic data, we consider a particular simulation yielding the same epidemic data as the 3-cluster epidemic. (a) Epidemiological parameters. (b) Evolutionary model parameters. (TIFF) [file pcbi.1004633.s003.tiff]

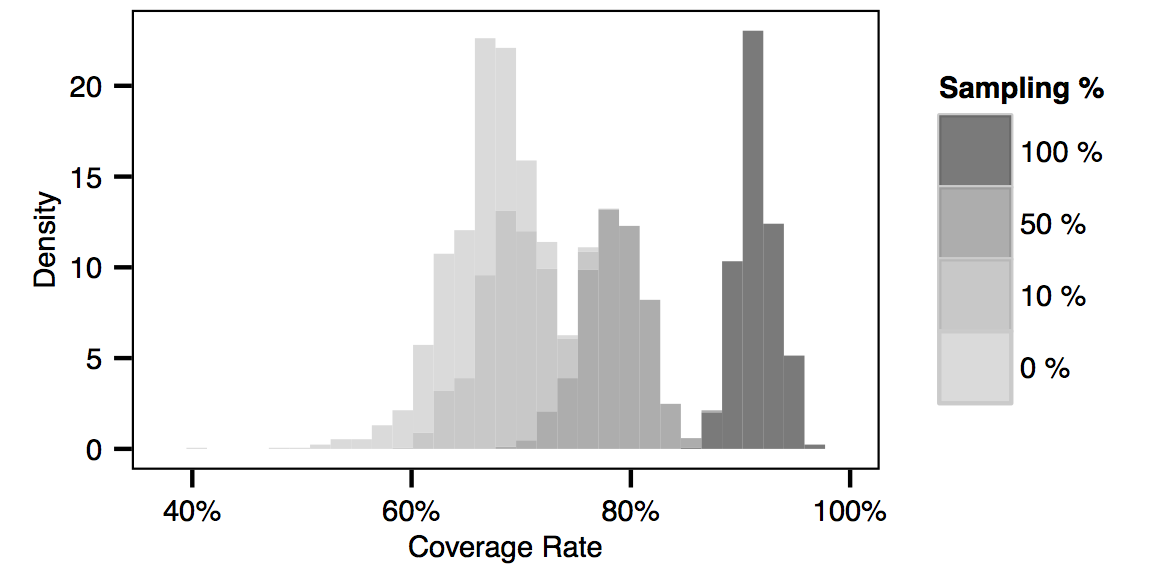

Supplement: S3 Fig — Posterior distributions of the overall coverage rate for the epidemic with lower mutation rates. Notice that, at the low sampling percentage (10%) the availability of genetic data may not increase significantly the coverage rates compared to the scenario without any samples. (TIFF) [file pcbi.1004633.s004.tiff]

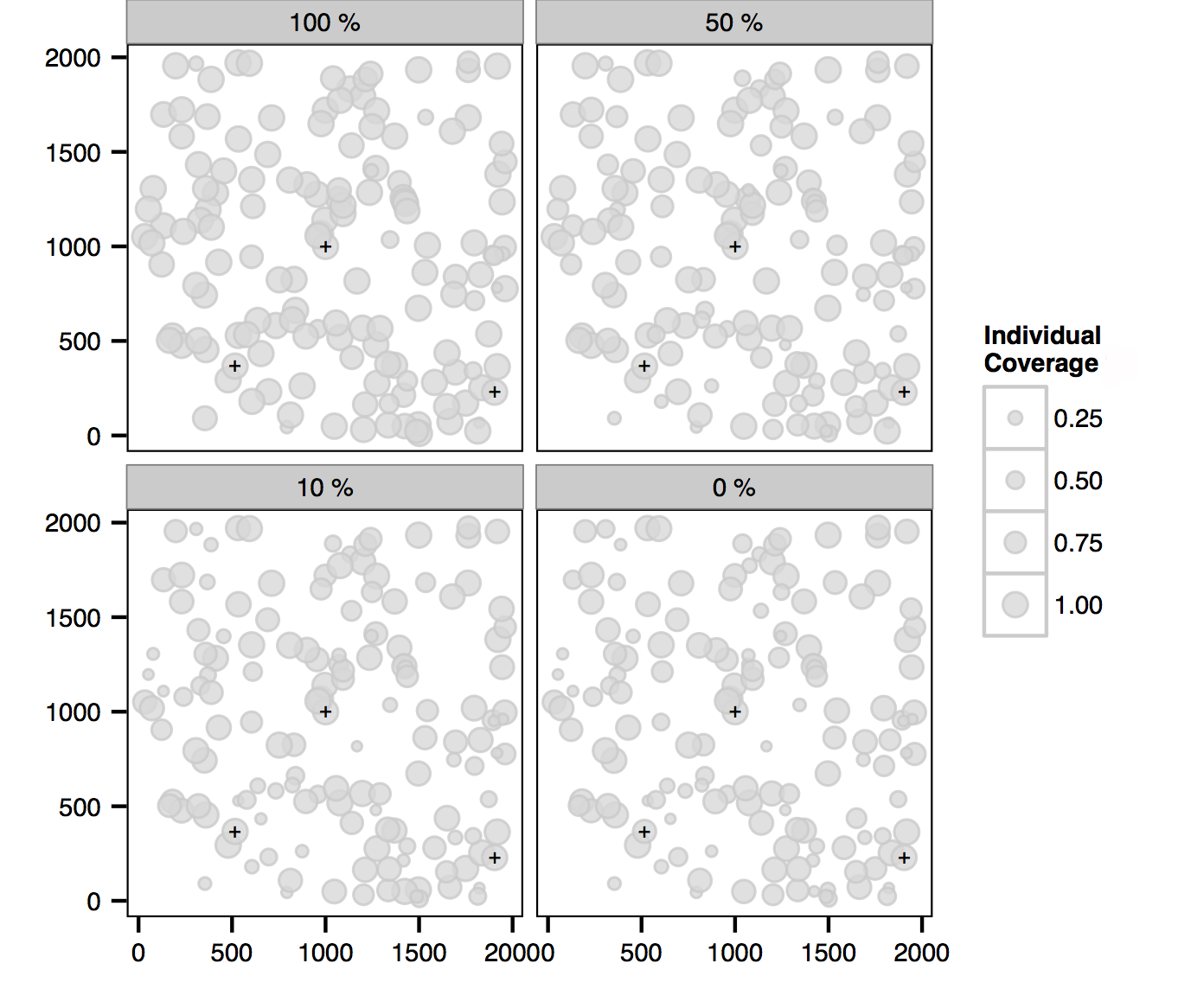

Supplement: S4 Fig — Posterior individual coverage of the sources of infection for the epidemic with lower mutation rates in scenarios with sampling 100%, 50%, 10% and 0%. The symbol + indicates an actual primary case. (TIFF) [file pcbi.1004633.s005.tiff]

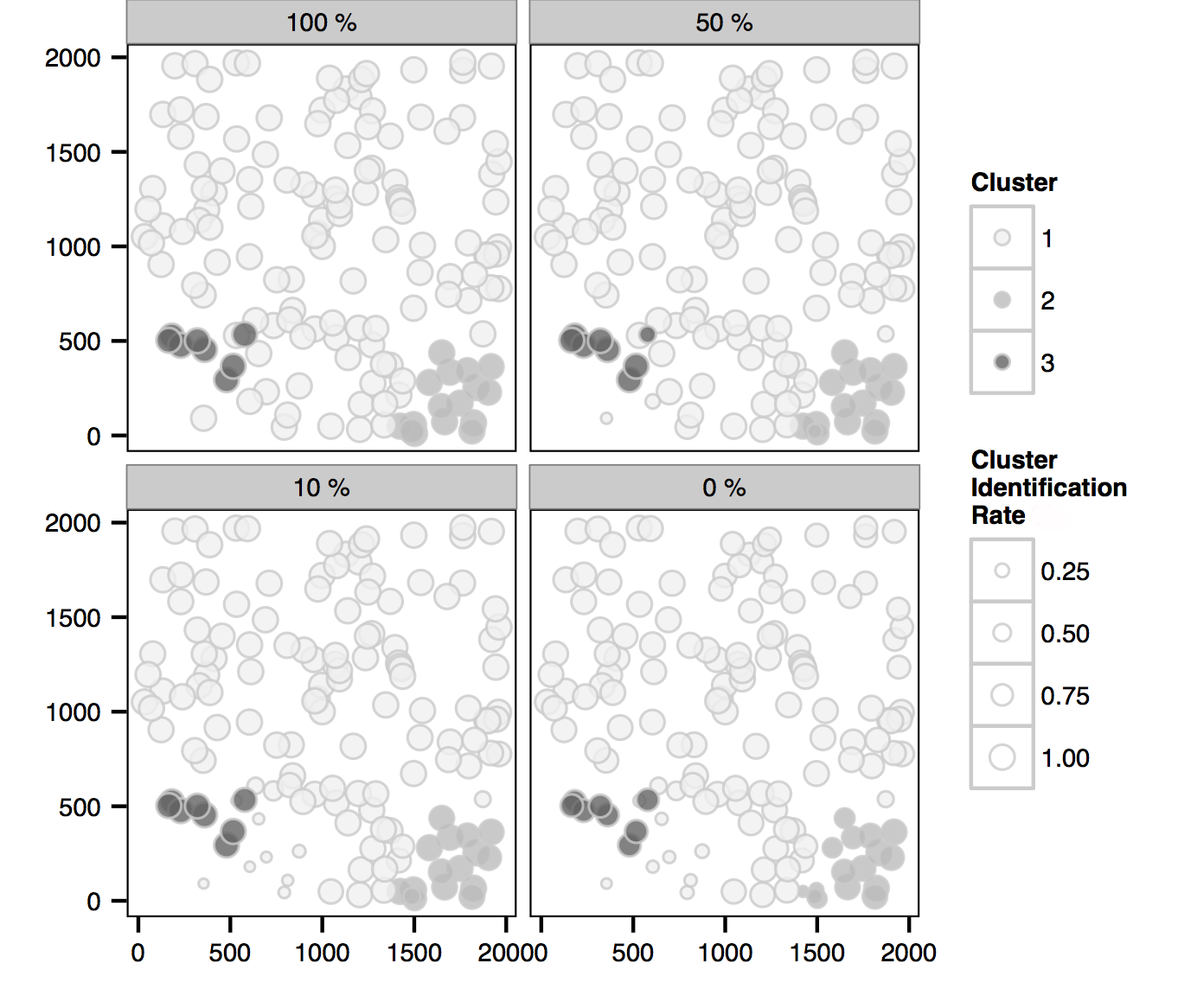

Supplement: S5 Fig — Posterior cluster identification rate of the infections (see definition in main text, within each actual cluster of the epidemic with lower mutation rates, in scenarios with sampling 100%, 50%, 10% and 0%. (TIFF) [file pcbi.1004633.s006.tiff]

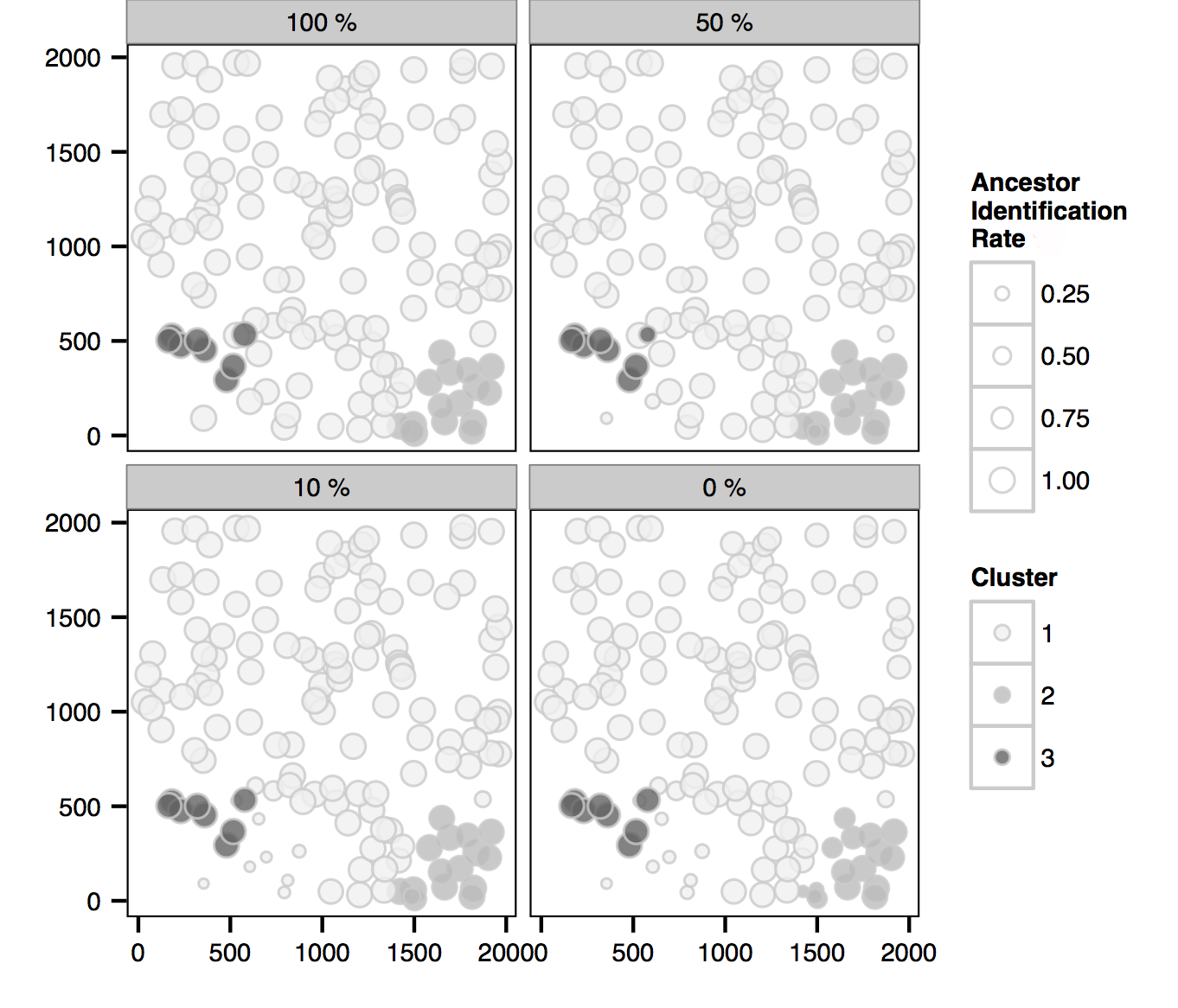

Supplement: S6 Fig — Posterior (primary) ancestor identification rate of the infections (see definition in main text), within each actual cluster of the epidemic with lower mutation rates, in scenarios with sampling 100%, 50%, 10% and 0%. (TIFF) [file pcbi.1004633.s007.tiff]

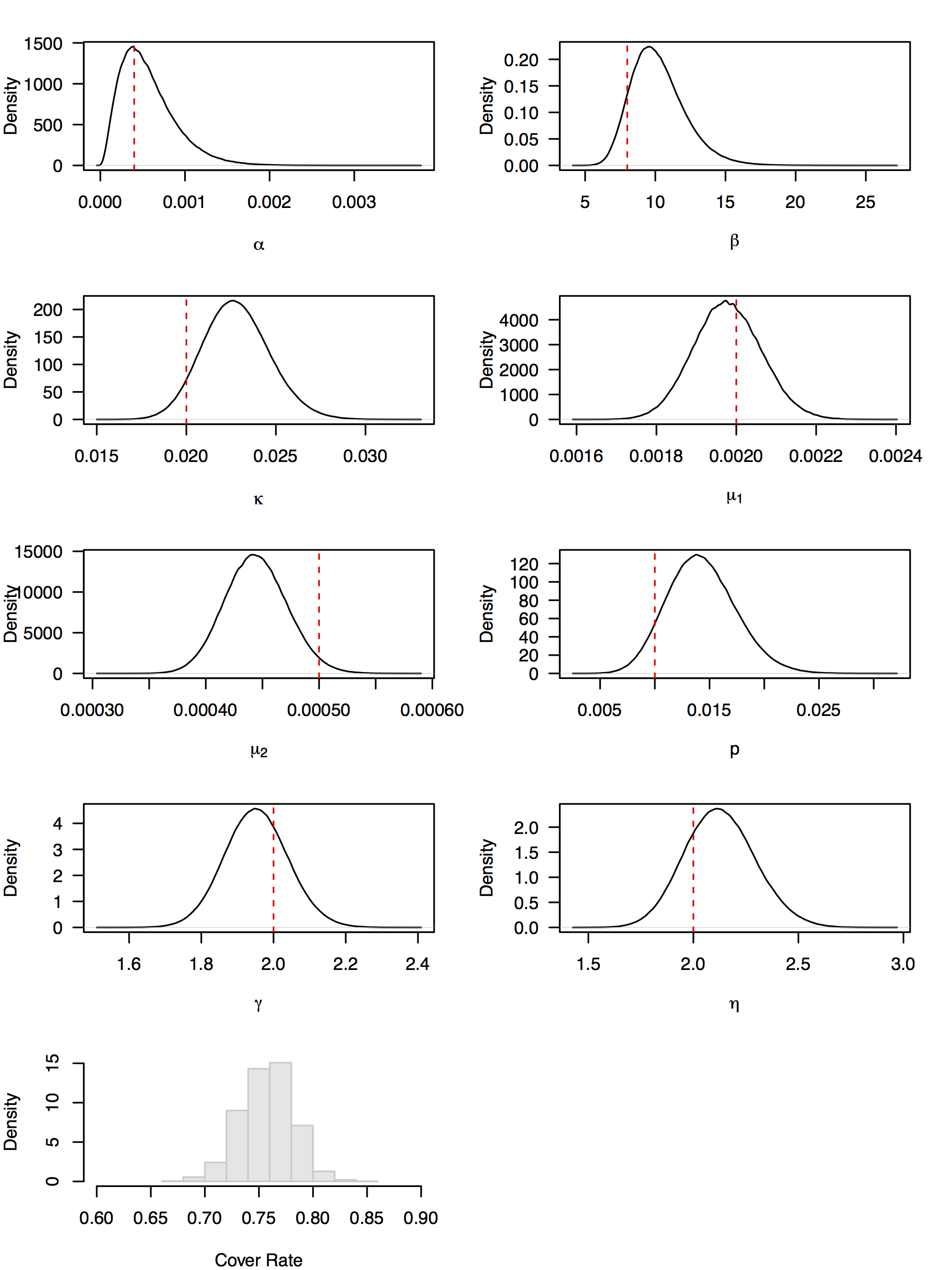

Supplement: S7 Fig — Posterior distributions of model parameters and the coverage rate from fitting the 3-cluster epidemic data with sampling proportion 20% (assuming the latent period distribution is known). (TIFF) [file pcbi.1004633.s008.tiff]

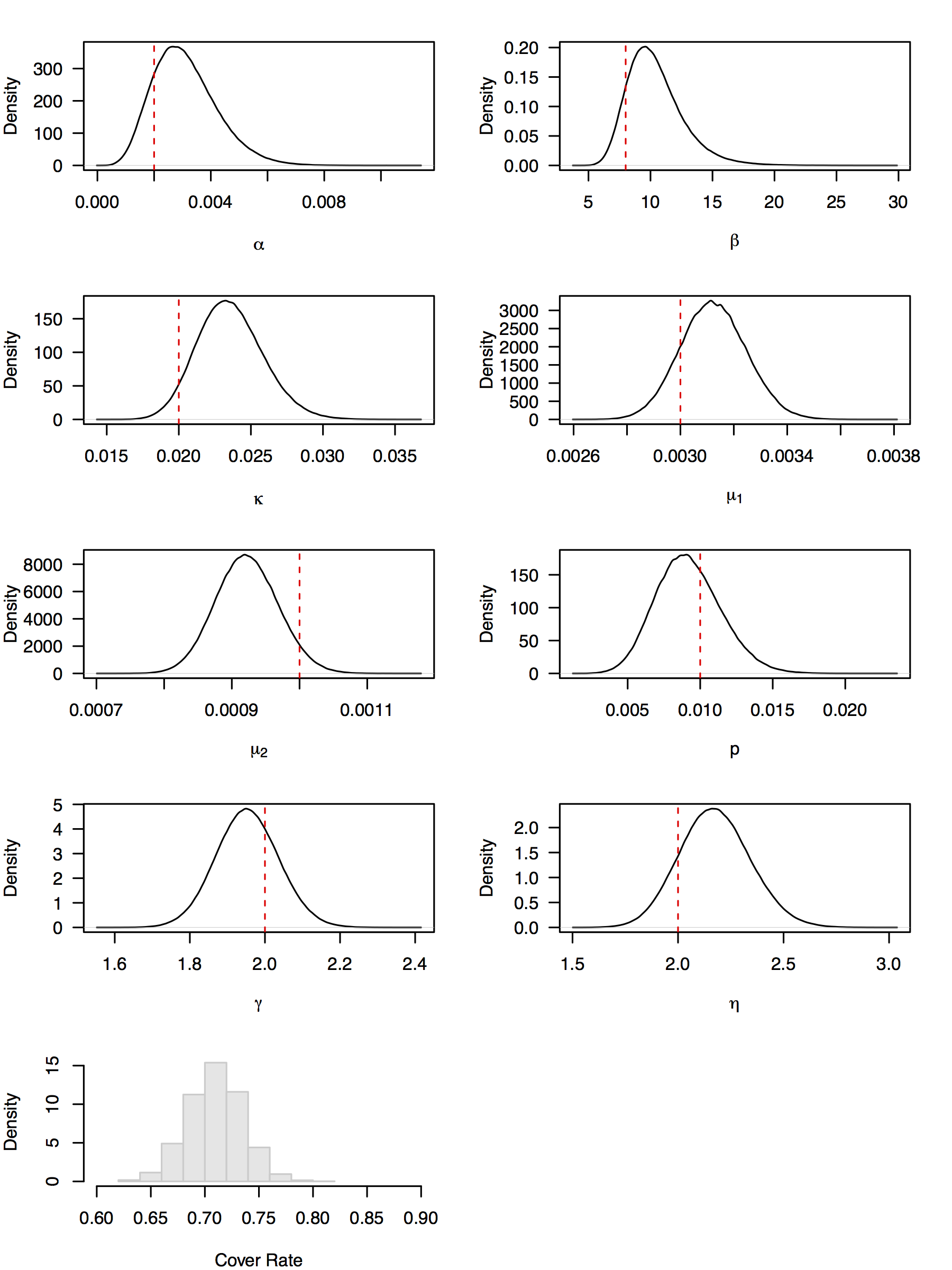

Supplement: S8 Fig — Posterior distributions of model parameters and the cover rate from fitting the 6-cluster epidemic data with sampling proportion 20% (assuming the latent period distribution is known). (TIFF) [file pcbi.1004633.s009.tiff]

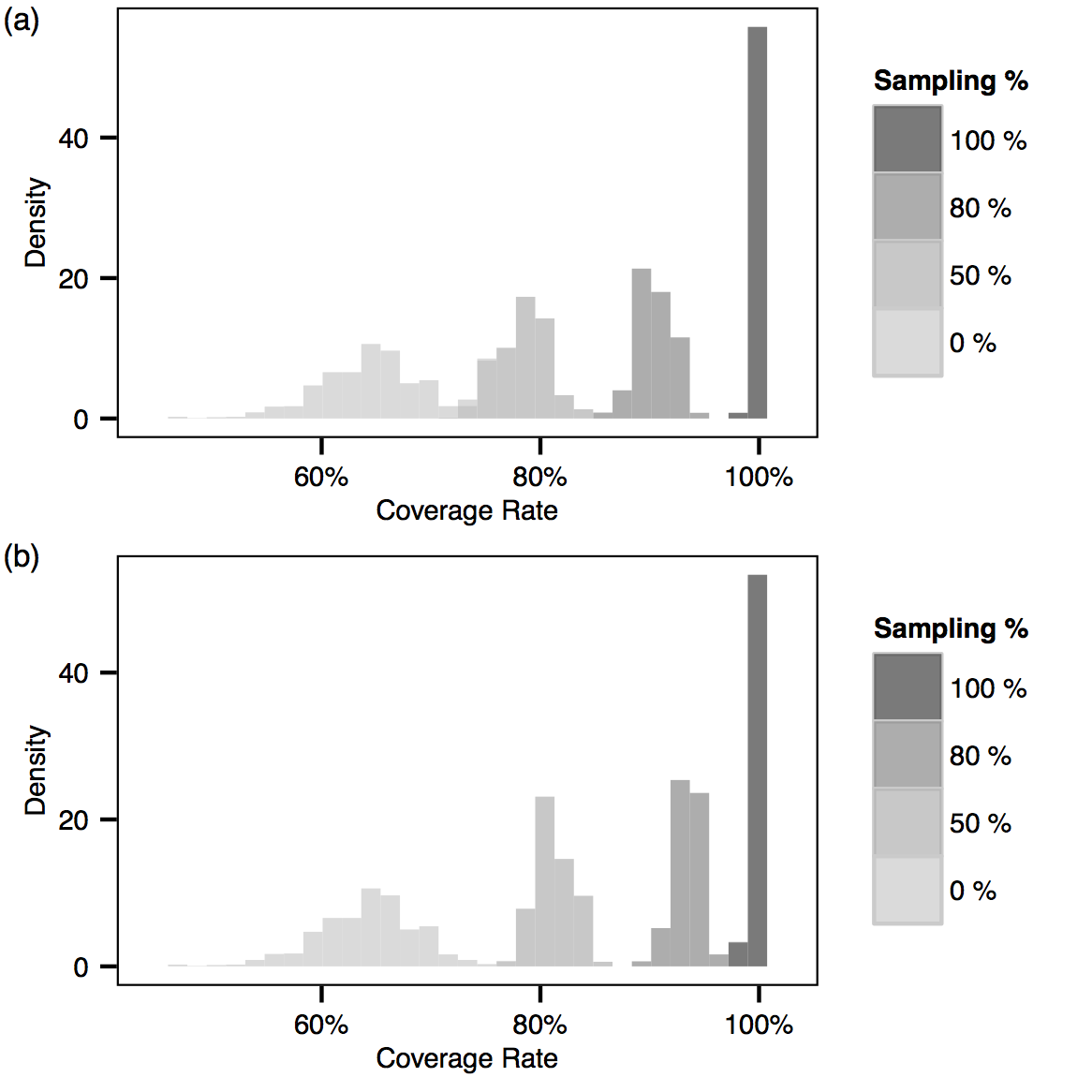

Supplement: S9 Fig — Posterior distributions of the overall coverage rate (with the single-cluster epidemic). (a) n = 1000. (b) n = 8000. We assume α = 0.0004, β = 10.0 and other parameters are the same as those used for simulating the 3-cluster epidemic i the main text. We consider a particular simulation giving rise to a single-cluster epidemic. (TIFF) [file pcbi.1004633.s010.tiff]

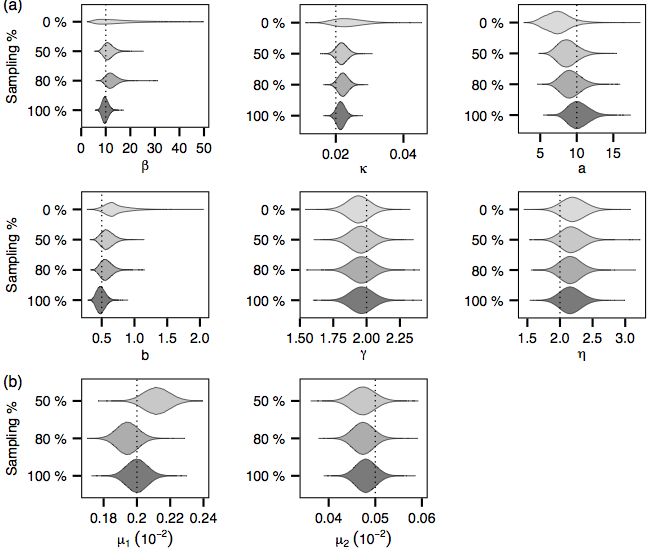

Supplement: S10 Fig — Violin plots showing the posterior distributions of the model parameters (with the single-cluster epidemic and number of bases n = 1000). Dashed lines represent the actual values of the model parameters. (a) Epidemiological parameters. (b) Evolutionary model parameters. (TIFF) [file pcbi.1004633.s011.tiff]

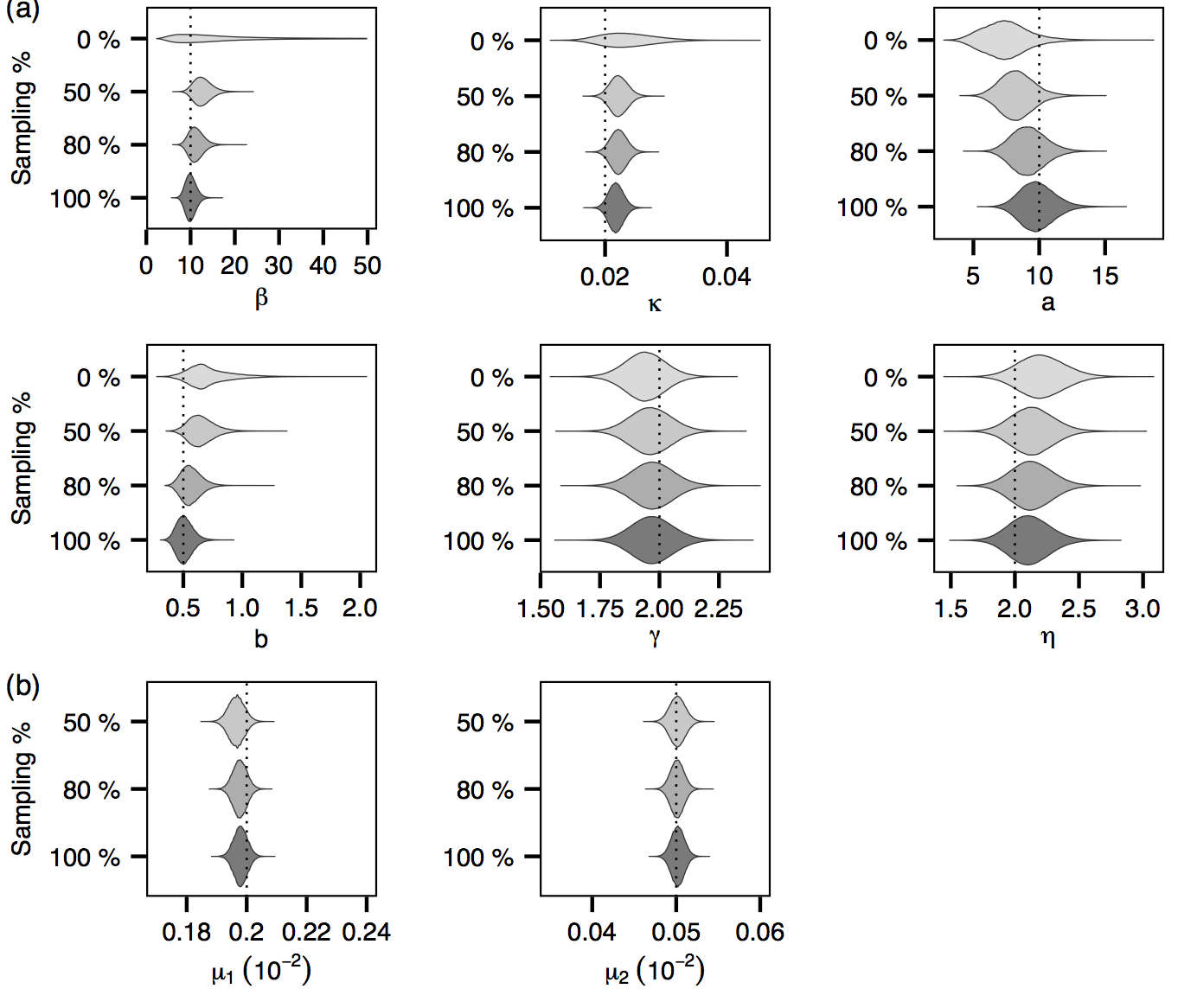

Supplement: S11 Fig — Posterior distributions of the model parameters (with the single-cluster epidemic and number of bases n = 8000). Dashed lines represent the actual values of the model parameters. (a) Epidemiological parameters. (b) Evolutionary model parameters. (TIFF) [file pcbi.1004633.s012.tiff]

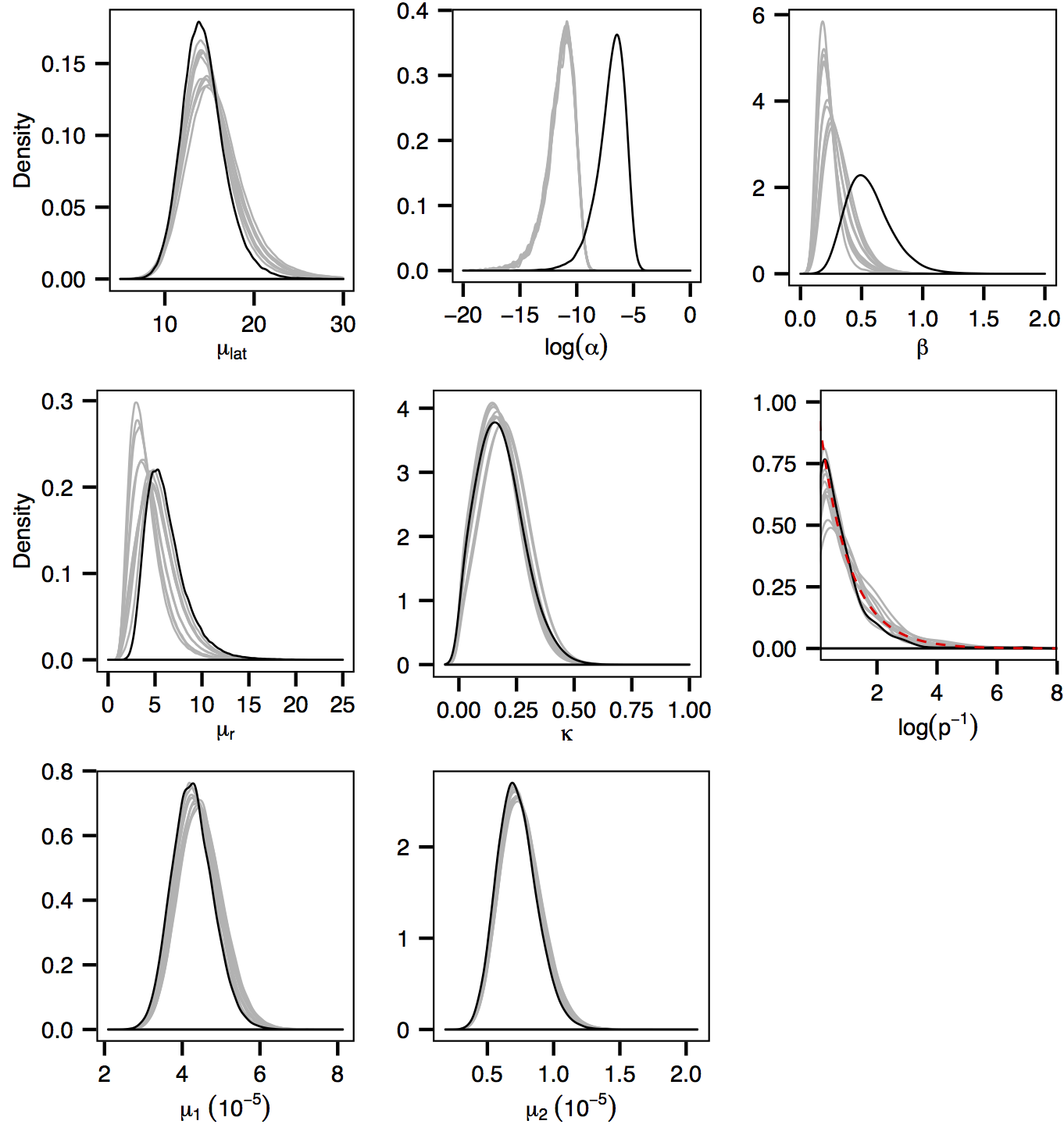

Supplement: S12 Fig — Posterior distributions of the full set of model parameters obtained from fitting the model to 10 independently simulated datasets obtained by adding 300 randomly assigned susceptible premises to the 2001 FMD data (grey curves). The posteriors corresponding to the case when susceptibles are not considered are coloured in black. Non-informative flat priors are used for model parameters. Note that the posterior distributions of p appear to be almost the same as the prior (i.e., U(0, 1)). To facilitate comparison, the posteriors of log(p −1) are presented and appear identical to an Exp(1)∼log(U(0, 1)−1) represented by the red dotted line, which suggests that the data are not sufficient for estimating p (see more discussion in S1 Text. (TIFF) [file pcbi.1004633.s013.tiff]
